# Genome-Wide Two-Component System Genes of Cytokinin Analysis and Their Functions of Drought Stress Response in *Pinus tabuliformis*

**DOI:** 10.3390/metabo16100741

**Published:** 2026-10-01

**Authors:** Hongmei Liu, Boning Yang, Jian Pan, Zhibing Wan, Lei Zheng

**Affiliations:** 1 College of Life and Environment Science, Huangshan University, Huangshan 245041, China; 600197@hsu.edu.cn (H.L.); panjian@hsu.edu.cn (J.P.); 2 The Key Laboratory of Forest Resources Conservation and Utilization in the Southwest Mountains of China Ministry of Education, Southwest Forestry University, No. 300 Bailongsi, Kunming 650224, China; yangboning@swfu.edu.cn

**Keywords:** *Pinus tabuliformis*, conifer, hormone, abiotic stress

## Abstract

Objective: Cytokinin signaling plays an important role in plant growth and development, but cytokinin two-component signaling system (TCS) genes have not been comprehensively reported in conifers. We aimed to identify and analyze the TCS family in *Pinus tabuliformis* and explore their roles in drought response. Methods: *PtTCS* genes were identified by BLASTP using Arabidopsis TCS sequences and validated by conserved domain analysis. Phylogenetic, structural, cis-element, GO enrichment, and expression profiling under drought were performed, with qRT-PCR validation. Results: Twenty-two *PtTCS* genes were identified (three *CHKs*, four *HPs*, eight *type-A RRs*, seven *type-B RRs*). Most proteins retained canonical domains, though some key residue variations were noted. Five genes (*PtCHK2*, *PtHP1*, *PtHP4*, *PtRR2*, *PtRR4*) were significantly upregulated under drought, opposite to the pattern in angiosperms, and rewatering restored expression. Conclusions: The drought-induced upregulation in *P. tabuliformis* contrasts with angiosperm downregulation, suggesting a distinct cytokinin-mediated drought response in conifers. This study provides the first comprehensive TCS inventory in a conifer and a foundation for functional studies in gymnosperms.

## 1. Introduction

Cytokinin (CK) as an important hormone, plays essential and crucial roles in various aspects of plant growth and development [1,2,3,4,5]. Cytokinin signaling is transducted by a two-component system (TCS) through a His-to-Asp phosphorelay [6,7]. Almost certainly the TCSs emerge from a bacterial two-component pathway, and this system is among the most widely distributed transduction systems, in both prokaryotic and eukaryotic organisms, which is one of the important signal transduction pathways for organisms to sense external environmental stimuli [8,9,10,11,12]. In plants, the TCS consists of a multistep phosphorelay [13,14]. Cytokinins are directly recognized by the CK receptor histidine kinase (CHK) at the plasma membrane, which results in autophosphorylation [6]. The phosphorylation signal is then transmitted to histidine phosphotransferase proteins (HPs), which are then transported to the nucleus. In the nucleus, HPs activate B-type response regulators (RRs) by phosphorylation, and they subsequently initiate the transcription of their target genes, including type-A RRs, a class of negative regulators of cytokinin signaling [1,15,16]. Through this multistep relay, *TCSs* play critical roles in transducing environmental inputs, including various abiotic stresses [12,17,18,19,20,21].

Recently, studies have shown that cytokinin signaling pathways respond immediately to drought stress [5,18,22,23,24,25]. In *Arabidopsis thaliana*, multiple TCS components function as negative regulators of drought stress, including the receptors *AHK2*, *AHK3*, and *AHK4* [26], the phosphotransmitters *AHP2*, *AHP3*, *AHP4*, and *AHP5* [18,27], and the B-type response regulators *ARR1*, *ARR10*, and *ARR12* [28,29]. Conversely, the type-A response regulator *ARR5*, a negative regulator of CK signaling, enhances plant drought resistance by inhibiting cytokinin signaling [25]. This pattern extends to other angiosperms: silencing the type-A RR gene *GhRR7* in *Gossypium* improves drought tolerance [30], and downregulation of the receptor gene *SlHK2* enhances drought tolerance in *Solanum lycopersicum* [19]. These results collectively support a model in which drought stress suppresses CK biosynthesis, and the resulting decrease in CK levels alleviates the inhibitory effect of CK signaling on stress-responsive genes, thereby promoting survival [5,31,32,33]. However, this model has been built almost exclusively on studies in angiosperms, leaving a critical knowledge gap for gymnosperms, particularly conifers. Angiosperms and gymnosperms diverged over 300 million years ago, and their long independent evolutionary histories mean that many molecular regulatory mechanisms characterized in angiosperms cannot be directly extrapolated to gymnosperms. This deep evolutionary divergence, together with the opposing physiological responses observed under drought, strongly suggests that the role and regulatory logic of CK signaling in drought resistance may fundamentally differ between conifers and angiosperms. Whether and how TCS components in conifers contribute to drought adaptation remains completely unaddressed. To date, there are few reports in coniferous trees, only the number of cytokinin members has been reported in *Picea abies* and *Pinus taeda* [9,34], and no in-depth study has been conducted. Recent research shows that the content of active CK in seedlings of *Pinus sylvestris* and *Picea abies* increases under drought conditions [35], contrasting with the drought-induced CK decline typical of angiosperms.

*Pinus tabuliformis* is an important indigenous species in China, which has excellent drought resistance, barren resistance and disease resistance, and is used as the ecological cornerstone tree species [36,37,38]. This species is widely distributed in the arid regions of central and northwestern China. Its water needs are heavily dependent on natural rainfall, making it particularly affected by drought stress [39]. Although a high-quality chromosome-level genome has recently become available for this species [40], the CK signaling gene family has not been systematically identified or analyzed, and its functional role in drought resistance remains entirely unknown. Here, we used *P. tabuliformis* as the experimental material, and a genome-wide identification of cytokinin signal pathway gene family members was performed. A systematic analysis of the gene characterization, gene structures, gene phylogeny and synteny was also conducted. Furthermore, expression profiles of those genes in different organs were analyzed. We also checked the expression profiles of those *TCS* genes under abiotic stress in form of drought stress. These results provide a foundation for further analysis of these gene functions.

## 2. Materials and Methods

### 2.1. Plant Materials

For tissue specific expression analysis, needles and hypocotyls were collected from *P. tabuliformis* seedlings that were cultured under control conditions (24 °C, 16 h light/8 h dark), and the sapling needles and shoots were sampled from 3-year-old trees. Adult tissue samples, including needles, shoots, stems, cones, and roots, were collected from a clonal seed orchard on 10 May 2016 (the orchard is located at the National Improved Variety Base of *Pinus tabuliformis*, Qigou Forest Farm, Pingquan City, Hebei Province, China; 118°44.6758′ E, 40°98.8784′ N, 560–580 m a.s.l.) [41,42]. All samples were quickly placed in liquid nitrogen and stored at −80 °C until total RNA extraction and RNA-seq analysis.

### 2.2. Identification of TCS Genes in P. tabuliformis

Genome sequences were obtained from the *P. tabuliformis* genome database [40]. The protein sequences of 24 *TCS* members of *Arabidopsis* were downloaded from the TAIR website (http://www.arabidopsis.org/) and then used as queries to perform BLASTP (Basic Local Alignment Search Tool: Protein BLAST) in the NCBI BLAST+ suite (version 2.16.0) searches from the *P. tabuliformis* genome with an E-value of 1 × 10^−6^ as the threshold. All putative PtTCS protein sequences were further confirmed according to whether they possessed the conserved domains of TCS proteins by searching Pfam database (http://pfam.janelia.org/; accessed on 6 May 2023).

### 2.3. Phylogenetic Analysis

The TCS protein sequences from *Physcomitrella patens*, *Populus trichocarpa*, Arabidopsis, and *P. tabuliformis* were used for phylogenetic analysis. Multiple sequence alignments of those proteins were performed using MUSCLE v3.8.31 with default parameters. A phylogenetic tree was subsequently constructed using the neighbor-joining method of MEGA-X, with the following parameters: p-distance model, partial deletion and 1000 bootstrap replications. The obtained phylogenetic tree was visualized by the online program Interactive Tree of Life v6 (iTOL) (https://itol.embl.de/) [43].

### 2.4. Gene Structure, Construction and Motif Analysis

Analysis of introns and exons was conducted using Gene Structure Display Server 2.04 (http://gsds.cbi.pku.edu.cn/). Motifs were identified using the online tool MEME Version 5.3.2 program (https://meme-suite.org/meme/tools/meme, accessed on 28 September 2026). The predicted peptide sequences of the conserved domain in the TCS proteins were identified using NCBI CCD online search (https://www.ncbi.nlm.nih.gov/Structure/bwrpsb/bwrpsb.cgi, accessed on 28 September 2026). TBtools-II (v2.x) was used to integrate and visualize phylogenetic trees, conserved motifs, domains, and gene structure results [44].

### 2.5. Chromosomal Localization and Subcellular Localization Prediction

Chromosome distribution information was retrieved from the *P. tabuliformis* genome database using TBtools software, and the results were visualized using the online tools MG2C v 2.1 (http://mg2c.iask.in/mg2c_v2.1/, accessed on 28 September 2026) [45]. The protein subcellular localization prediction used the online tools at WoLF PSORT (https://wolfpsort.hgc.jp/, accessed on 28 September 2026).

### 2.6. Collinearity Analysis Between P. tabuliformis and Other Gymnosperms

The collinearity analysis of *PtTCS* genes was done using JCVI v0.84 software, which identified syntenic gene pairs between *P. tabuliformis*, *Ginkgo biloba* and *Sequoiadendron giganteum* [46]. The result was visualized with the TBtools software [44].

### 2.7. Cis-Elements Analysis and GO Analysis

The upstream sequences (2 kb) from the transcriptional start site of each gene were extracted from the *P. tabuliformis* genome database. The cis-elements were analyzed in the PlantCARE program (http://bioinformatics.psb.ugent.be/webtools/plantcare/html/, accessed on 28 September 2026). The results were visualized with TBtools software [44]. Furthermore, the Cytoscape 3.9.1 ClueGo tool was used to analyze the Gene Ontology.

### 2.8. Transcriptome Analysis and Expression of Cytokinin Pathway Genes

Total RNA was extracted from different *P. tabuliformis* samples using TRIZOL reagent (Invitrogen, Karlsruhe, Germany). The cDNA library was constructed using Illumina TruseqTM RNA sample prep Kit (Tiangen Biotech (Beijing) Co., Ltd., Beijing, China). The Illumina Novaseq platform (2 × 150 bp) was used for transcriptome sequencing. Clean reads for each sample were aligned to the *P. tabuliformis* reference transcriptome. The Transcripts Per Million reads (TPM) algorithm was used for quantification of gene expression. Differential expression analysis was performed by Sleuth. The drought transcriptome data came from Niu lab [39].

### 2.9. The qPCR Validation

To validate the RNA-seq data, qRT-PCR was performed on differentially expressed genes. The seedlings samples from the short-term drought (8 days), long-term drought (23 days) and the control groups were collected and immediately placed in liquid nitrogen and stored at −80 °C.

## 3. Results

### 3.1. Identification of the TCS Genes in P. tabuliformis

A total of 22 putative PtTCS protein sequences were identified in the *P. tabuliformis* genome database [40] by employing 29 related protein sequences (Tables S1 and S2) from Arabidopsis as queries. With the online tools Pfam and NCBI CDD, the existence of typical conserved domains was detected (Figures S1 and S2). Based on the domain architecture, these 22 *PtTCS* genes were divided into three groups: three *CHKs*, four *HPs* and fifteen *RRs*. The basic information of all *PtTCS* genes was generated (Table 1), including the gene name, gene ID, amino acid length, molecular weights (MW), isoelectric points (pI) value and the grand average of hydropathicity (GRAVY). The results showed that the amino acid length of those PtTCS peptides ranged from 151 (PtHP1 and PtHP4) to 1063 (PtCHK3), and the MW varied from 17,393.74 (PtHP4) to 116,726.51 (PtCHK3) Da. The pI value ranged from 4.81 (PtHP4) to 7.82 (PtCHK3). All GRAVYs were negative, indicating that all 22 PtTCS proteins were hydrophilic.

### 3.2. Gene Structure, Conserved Domain and Motifs Characterization of PtTCS

**Figure 1:**
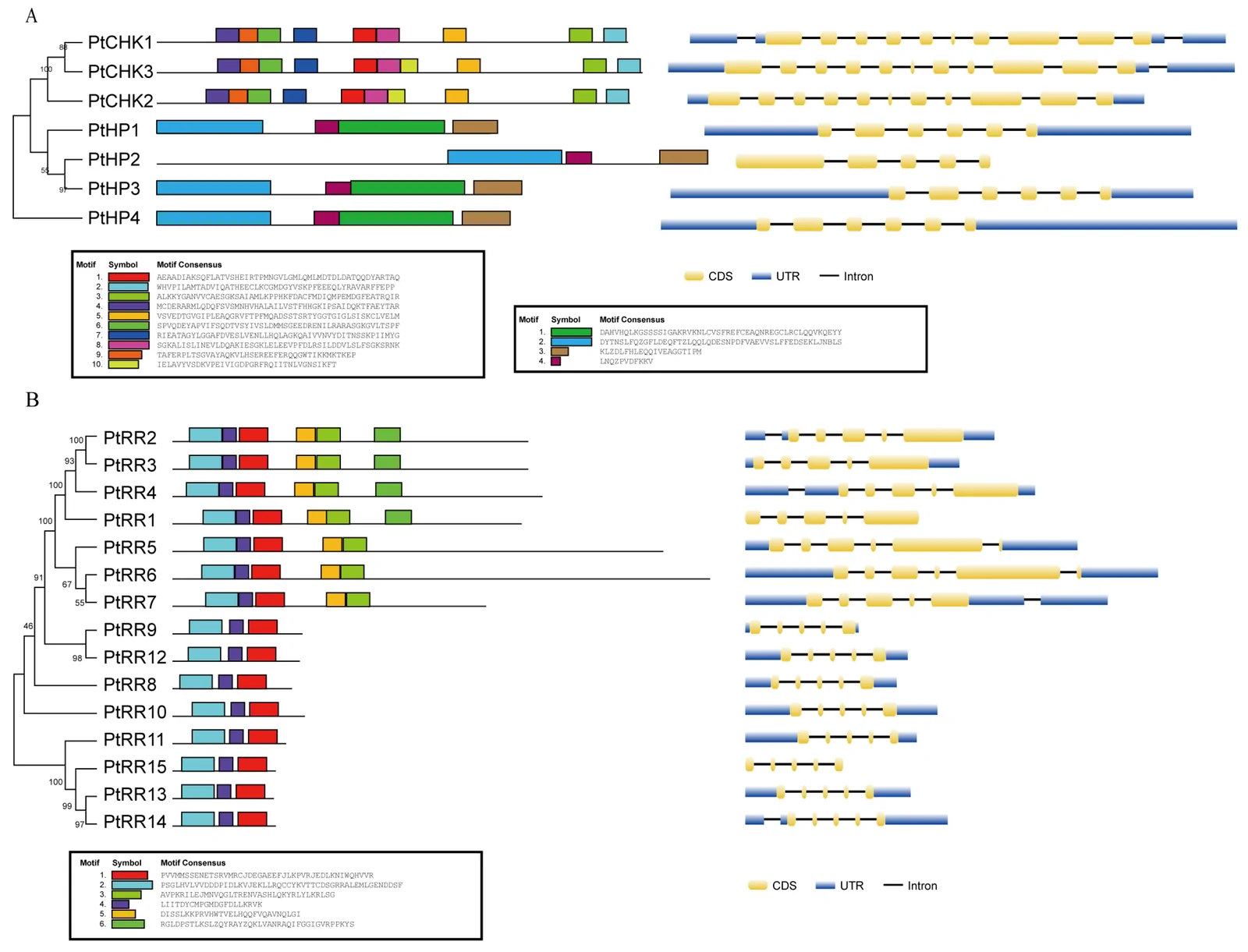
Conserved domain and gene structure of *PtCHKs*, *PtHPs* and *PtRRs* from *Pinus tabuliformis*. (**A**) Conserved domain and gene structure of *PtCHKs* and *PtHPs*; (**B**) Conserved domain and gene structure of *PtRRs*.

Conserved motifs and domains across the 22 PtTCS proteins were identified using MEME and NCBI CDD/Pfam analyses (Table S2). Among the eight putative HKs, only three contain the characteristic CHASE, HisKA, HATPase_c, and REC domains and were therefore classified as cytokinin receptors (PtCHK1–3). These three PtCHKs possess nine to ten conserved motifs (Figure 1A and Figure S1A). Eight HP proteins were identified, four of which harbor the complete Hpt domain and were designated authentic HPs (PtHP1–4), while the remaining four were considered pseudo HPs. All authentic PtHPs except PtHP2 contain four conserved motifs (motifs 1–4); PtHP2 lacks motif 1 but retains the intact Hpt domain with the signature XHQXKGSSXS motif (Figure 1A and Figure S1B). A total of 15 response regulator (RR) proteins were predicted. Based on domain architecture and motif composition, eight were classified as type-A RRs, each containing a single REC domain and a variable C-terminal extension with three conserved motifs (Figure 1B and Figure S2). Seven type-B RRs were identified, each possessing an N-terminal REC domain and a C-terminal Myb-like GARP DNA-binding domain. All type-B RRs share five conserved motifs (motifs 1–5), with PtRR5–7 additionally containing motif 6 (Figure S3).

Analysis of gene structures revealed that the three *PtCHK* genes contain 10–11 introns, whereas the four *PtHP* genes have four to six introns (Figure 1A). The type-A *PtRR* genes generally possess four introns, except *PtRR14*, which contains five; most type-B *PtRR* genes contain four or five introns, with the exceptions of *PtRR8* and *PtRR9*, which have 11 and 12 introns, respectively (Figure 1B).

### 3.3. Phylogenetic Analysis of PtTCS Proteins

**Figure 2:**
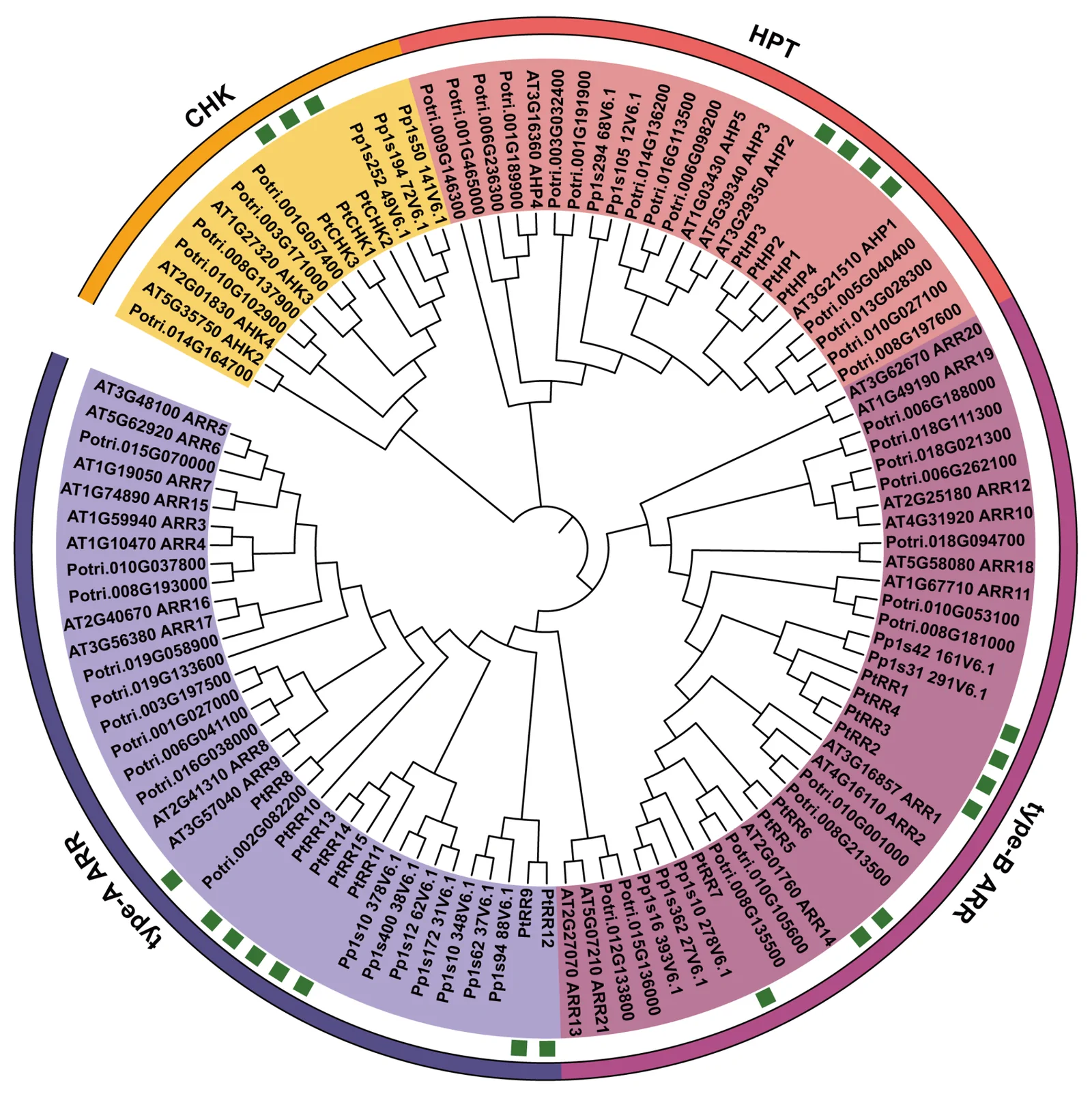
Phylogenetic analysis of cytokinin signal transduction pathway genes in *Physcomitrella patens*, *Populus trichocarpa*, *Arabidopsis thaliana* and *Pinus tabuliformis*. The green boxes indicate the members from *Pinus tabuliformis*.

A neighbor-joining phylogenetic tree was constructed based on the full-length protein sequences of 110 TCS members from *P. patens*, *P. trichocarpa*, Arabidopsis, and *P. tabuliformis* (Figure 2, Table S2). The proteins were resolved into four major groups: CHK (14), HP (25), type-A RR (39), and type-B RR (32).

The CHK proteins grouped into four distinct clades. All three PtCHKs clustered with three CHKs from *P. patens* in a clade that was separate from the angiosperm CHKs. The four authentic PtHP proteins formed a single cluster and showed the highest similarity to AHP1. The RR proteins were clearly divided into type-A and type-B subfamilies, consistent with the conserved domain analysis. The eight type-A PtRRs grouped together with seven type-A RRs from *P. patens*, while the seven type-B PtRRs were distributed across multiple clades alongside type-B RRs from the other three species.

### 3.4. Chromosome Distribution and Subcellular Location Analysis

The 22 *PtTCS* genes were mapped to 10 of the 12 *P. tabuliformis* chromosomes, and Chr2 and Chr7 harbored no *PtTCS* members (Figure S4). Three *PtCHK* genes were mapped on Chr6, Chr9 and Chr10, and four *PtHP* genes were located on Chr1, Chr10 and Chr12. The *PtRR* genes were unevenly mapped on all *P. tabuliformis* chromosomes, being absent from Chr1, Chr2, Chr7, and Chr10.

Subcellular localization predicted by WoLF PSORT (Table S4) indicated that three PtCHK proteins were located in the plasma membrane. PtHP1 and PtHP2 were located in the cytoplasm, PtHP3 is located in the mitochondria, and PtHP4 may be present in the chloroplast. One-half of type-A ARR proteins were located in the nucleus, including PtRR8, PtRR10, PtRR11, and PtRR15. The remaining four type-A ARR were localized to the cytoplasm. Most of the type-B ARR proteins were predicted to locate in the nucleus, except for PtRR1, which was located in the chloroplast.

### 3.5. Collinearity Analysis of PtTCSs

**Figure 3:**
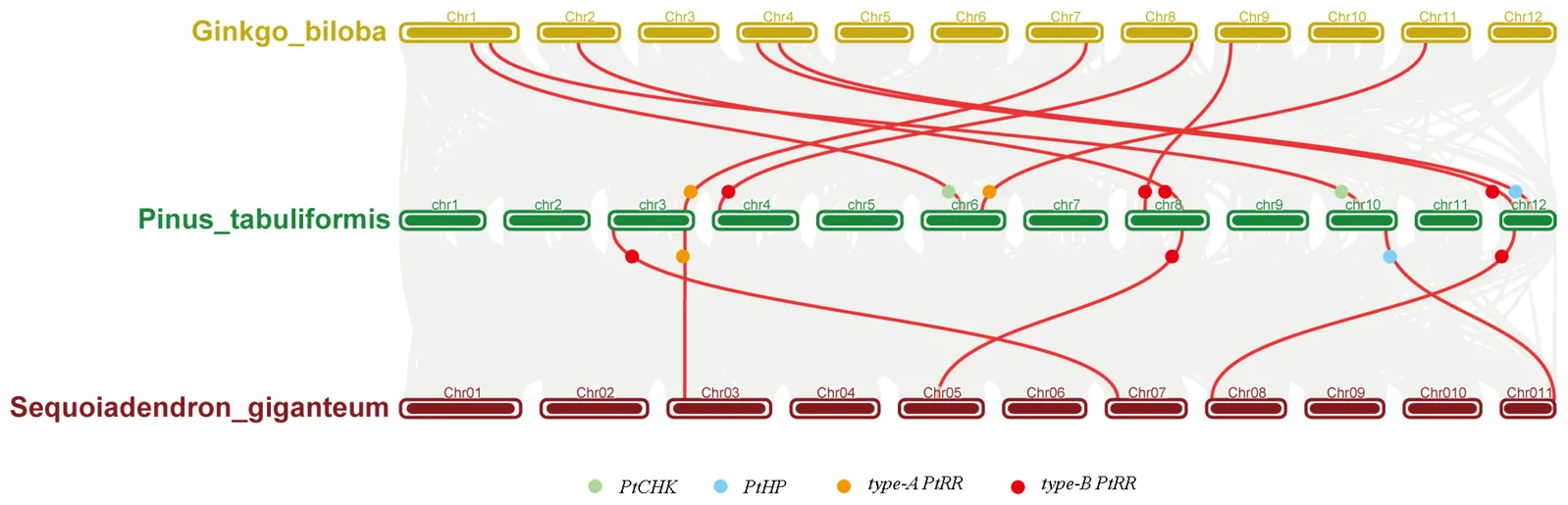
Collinearity analyses of *TCS* genes between *Pinus tabuliformis*, *Ginkgo biloba* and *Sequoiadendron giganteum*. Gray lines in the background represent collinear blocks in *P. tabuliformis*, *G. biloba* and *S. giganteum* genomes. The collinear gene pairs with *PtTCS* genes between *G. biloba* and *S. giganteum* are highlighted by the red lines.

To further investigate the evolutionary relationship and homology of *TCS* members, we performed a comparative collinearity analysis of the *PtTCS* genes with those of *G. biloba* and *S. giganteum* (Figure 3). A total of 14,766 collinear gene pairs were identified between *P. tabuliformis* and *G. biloba* and 41,551 between *P. tabuliformis* and *S. giganteum*. Within the *TCS* gene family, nine *PtTCS* genes showed collinearity with *G. biloba* orthologs and five with *S. giganteum* orthologs. The majority of these collinear *PtTCS* genes belonged to the type-B *PtRR* group. Among them, one type-A *PtRR* (*PtRR10*) and two type-B *PtRRs* (*PtRR4* and *PtRR7*) had orthologs in both species.

### 3.6. Analysis of Cis-Elements in the PtTCS Genes Promoters

**Figure 4:**
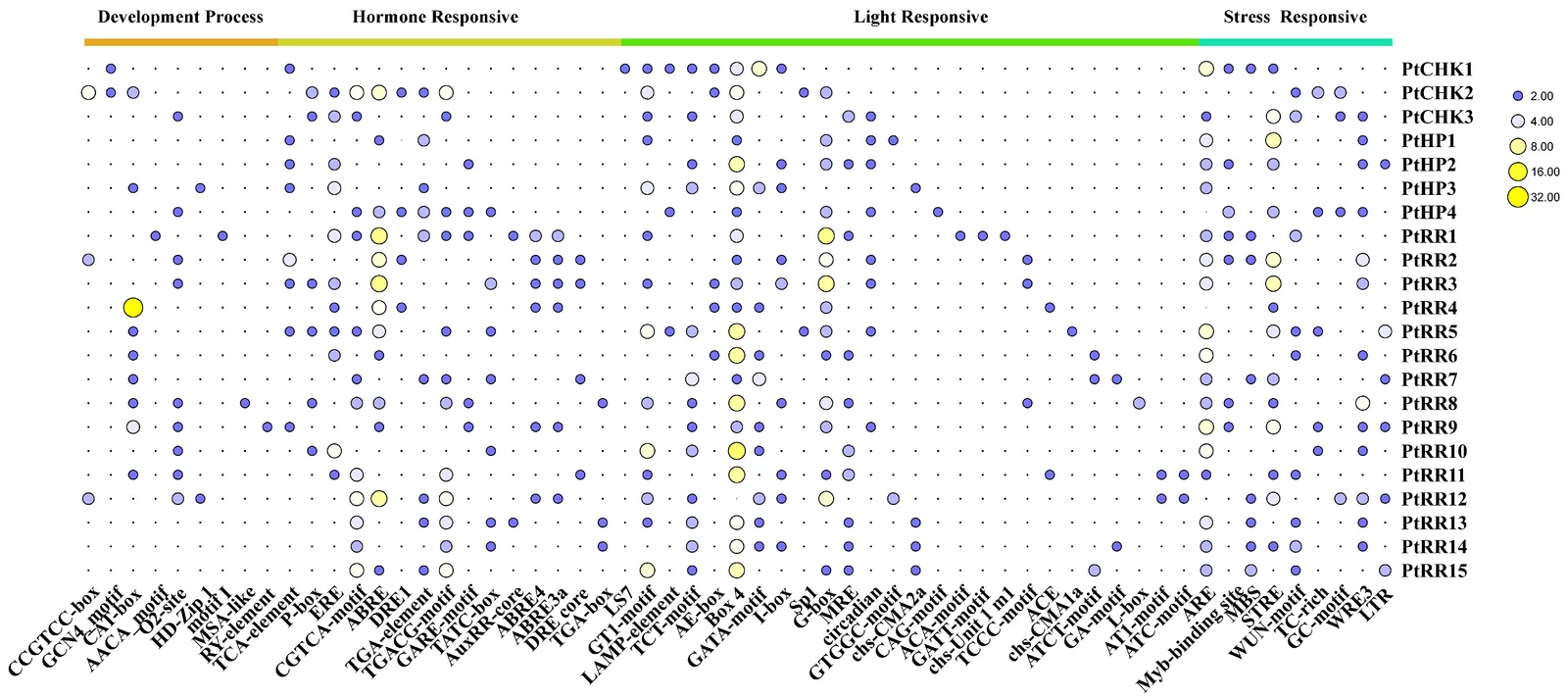
Heat map of cis-elements in the promoter region of *PtTCS* genes.

Analysis of the 2000 bp promoter regions upstream of the start codon identified 59 distinct cis-acting elements, which were grouped into four functional categories: hormone-responsive (15 elements), light-responsive (26), stress-responsive (9), and development-related (9) (Figure 4, Table S5). Nearly all promoters contained elements from all four categories, with individual promoters harboring multiple elements.

Light-responsive elements constituted the largest proportion. Among them, GT1-motif, TCT-motif, G-box, and Box 4 were distributed across almost all promoters. Stress-responsive elements identified included LTR (low-temperature), MBS (drought-inducible), ARE (anaerobic induction), and WUN-motif (wound-responsive). Development-related elements, such as CAT-box (meristem expression), O2-site (zein metabolism regulation), RY-element (seed-specific regulation), and endosperm-related motifs (GCN4_motif, AACA_motif), were also detected. In addition, hormone-responsive elements were present in all gene families, including ABRE (abscisic acid), P-box and TATC-box (gibberellin), TGA-element and TGA-box (auxin), TCA-element (salicylic acid), and CGTCA-motif (MeJA).

### 3.7. GO Enrichment Analysis of PtTCS Genes

**Figure 5:**
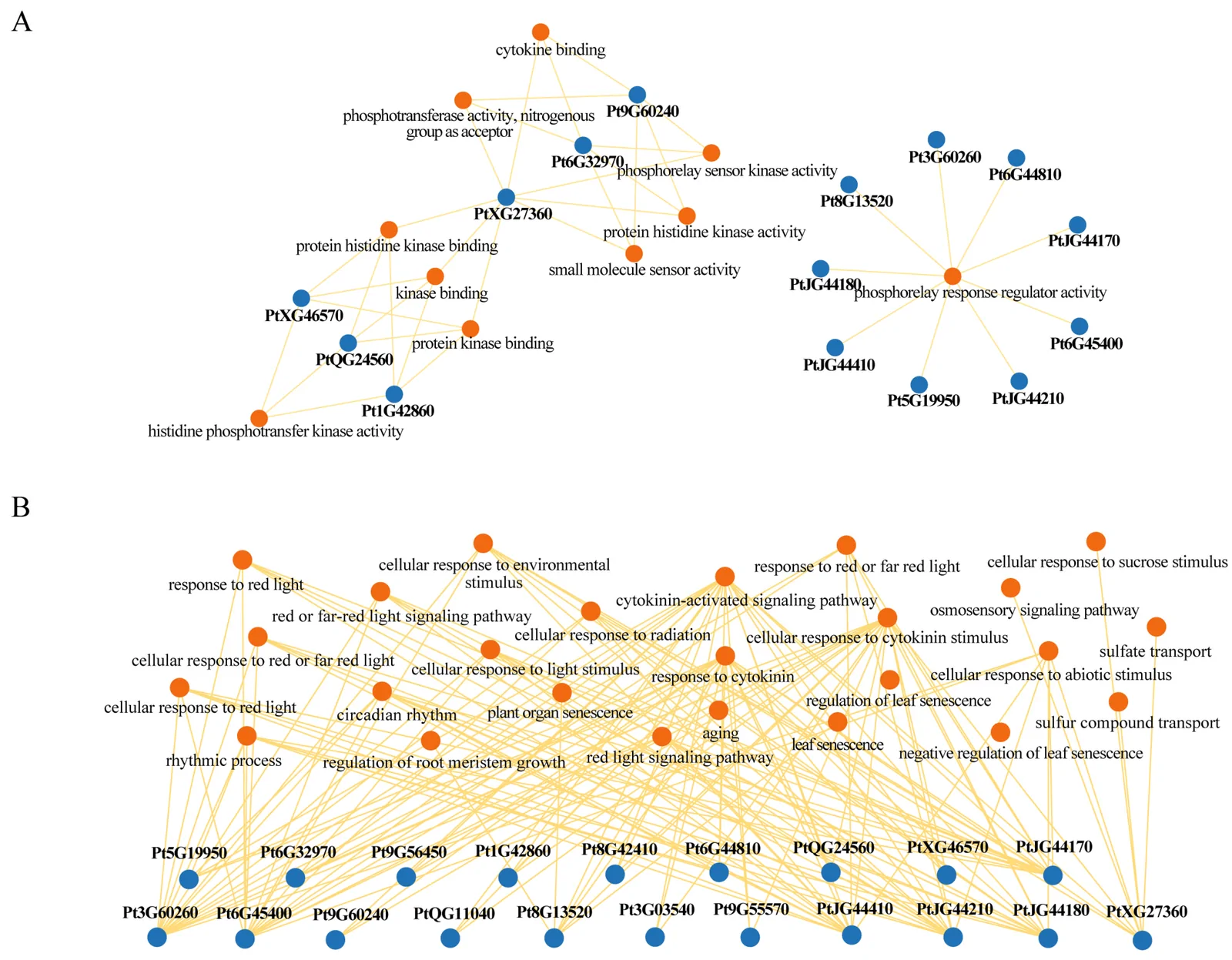
GO analysis of *PtTCS* genes. (**A**) Biological processes and (**B**) molecular functions. Blue circles represent genes, and orange circles show the process involved.

GO enrichment analysis assigned the *PtTCS* genes to biological processes (BP) and molecular functions (MF) categories (Figure 5). Within BP, three *PtCHK* genes were associated with cytokinin binding and kinase activity. Three *PtHP* genes (except *PtHP3*) were identified to have a role in kinase binding. All type-A *PtRR* genes were associated with phosphorelay response regulator activity. Within MF, the *PtTCS* genes were associated with cytokinin signaling, and several members were also annotated with terms related to responses to environmental stimuli.

### 3.8. Expression Profiles of PtTCS Genes in Various P. tabuliformis Organs

**Figure 6:**
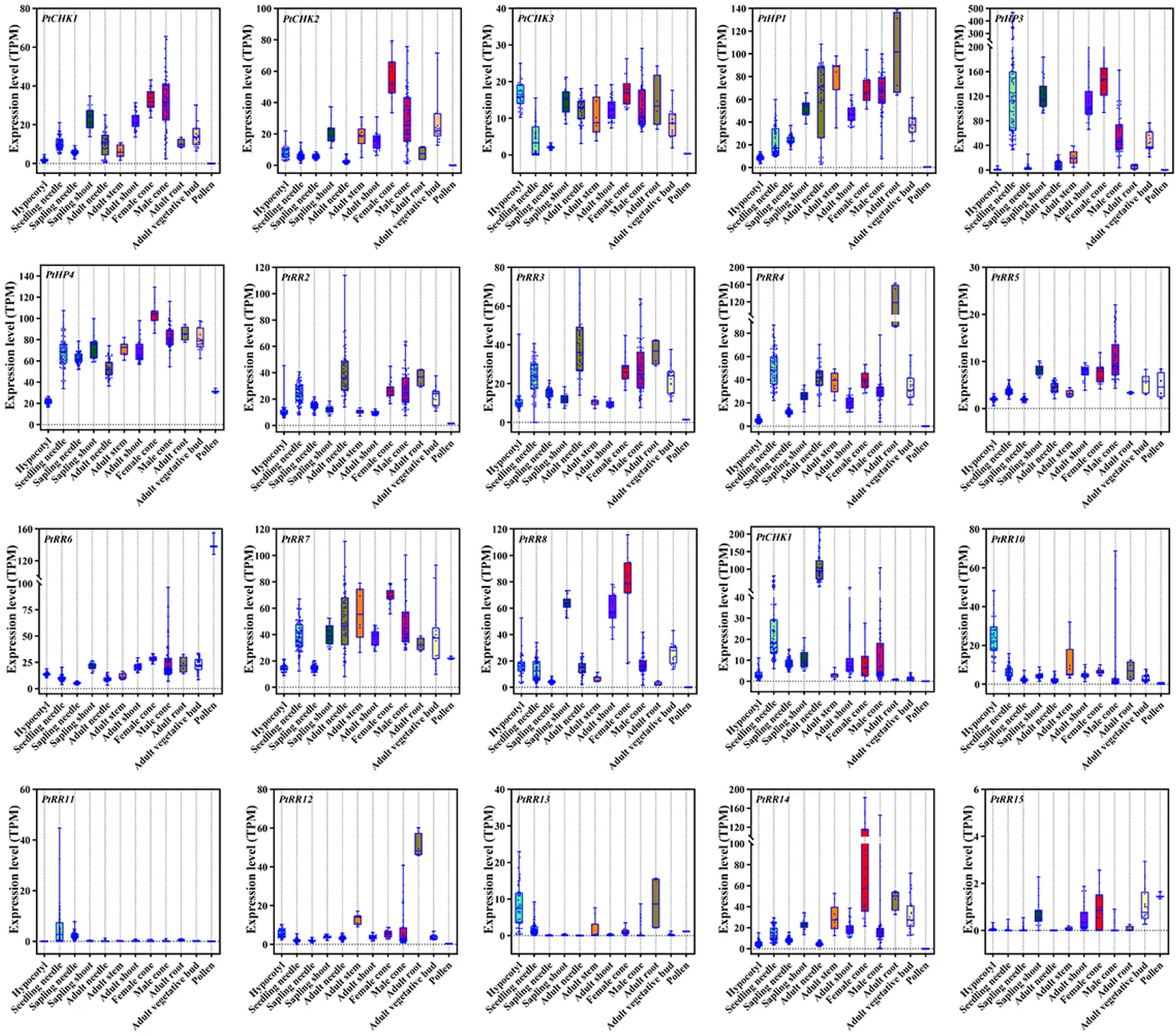
*PtTCS* genes expression profiles in various organs of *Pinus tabuliformis*. The expression levels of all *PtTCS* in 403 total samples of *P. tabuliformis* were analyzed through RNA-seq, including samples of the hypocotyl (2 months of age, *n* = 45), seedling needles (2 months of age, *n* = 90), sapling needles (3–5 years of age, *n* = 60), sapling shoots (3–5 years of age, *n* = 18), adult needles (33 years of age, *n* = 48), adult stems (33 years of age, *n* = 9), adult shoots (22–33 years of age, *n* = 30), female cones (33 years of age, *n* = 18), male cones (33 years of age, *n* = 57), adult roots (33 years of age, *n* = 4), adult vegetative bud (33 years of age, *n* = 21) and pollen (33 years of age, *n* = 3). The lower box and upper boundaries indicate the 25th and 75th percentiles for each data set. The horizontal lines inside the boxes represent the median values. Whiskers indicate the maximum and minimum values.

The expression patterns of *PtTCS* genes were examined across needles, stems, shoots, roots, vegetative buds, male and female flower buds, and pollen using available transcriptome data. All *PtTCS* genes were detected except *PtHP2* (Figure 6). Three cytokinin receptor *PtCHK* genes were highly expressed in all organs except pollen. Among the three *PtHPs* detected, only *PtHP4* was expressed in pollen. The expression of 15 PtRR genes in various organs was different. Eight *PtRR* genes were expressed in male and female cones, and *PtRR1*, *PtRR3*, *PtRR4*, *PtRR11*, and *PtRR13–15* were not detected in needles. *PtRR6* and *PtRR15* were highly expressed in pollen, which may be involved in pollen development. *PtRR2*, *PtRR4*, *PtRR10*, and *PtRR12* were expressed in roots.

### 3.9. Expression Profiling of PtTCS Genes During Drought Stress

**Figure 7:**
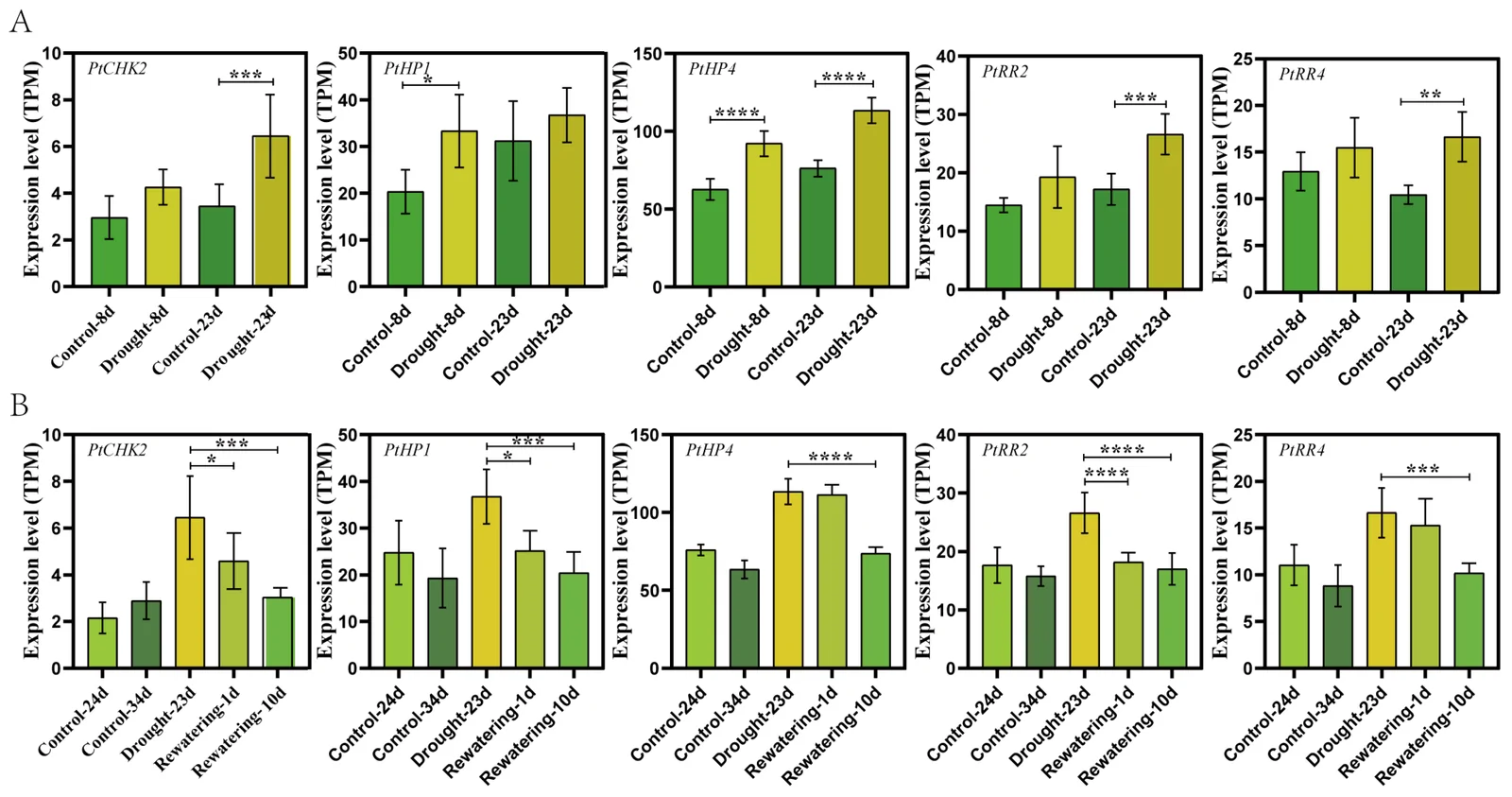
Expression patterns of *PtTCS* genes under drought (**A**) and rewatering (**B**). Asterisks indicate significant differences: * *p* < 0.05, ** *p* < 0.01, *** *p* < 0.001, **** *p* < 0.0001.

Based on the expression patterns of the 22 *PtTCS* genes in different organs, some genes were found to have high expression levels in roots, and their expression during different drought treatment times was further detected. The results showed that five genes showed significant differences under drought stress including one *PtCHKs*, two *PtHPs* and two type-B *PtRRs* (Figure 7A). *PtCHK1* and *PtCHK2* genes showed opposite change patterns during drought stress. Under the drought condition, the expression of *PtCHK1* was inhibited, while the expression of *PtCHK2* was induced. In short-term drought (8 days), neither *PtCHK1* nor *PtCHK2* showed significant changes, but in long-term drought (23 days), both showed significant changes. The expression of *PtHP1* and *PtHP4* were upregulated by drought, and the upregulation of *PtHP4* was extremely significant. *PtHP1* only showed significant upregulation in short-term drought. *PtRR2* and *PtRR4* acted as the primary cytokinin response regulators, which had similar expression pattern, neither changed significantly under short-term drought, while both were significantly upregulated under long-term drought.

### 3.10. Restoration of PtTCS Genes Expression During Re-Watering

Plants subjected to 23 days of drought were rewatered, and the expression of the five drought responsive genes were detected on days 1 and 10 after rehydration. The results showed that rewatering reduced the expression of upregulated genes under drought conditions, and the expression levels of these genes on the tenth day were similar to those of the control group (control-34d) (Figure 7B).

### 3.11. Validation of Transcripts by qRT-PCR

**Figure 8:**
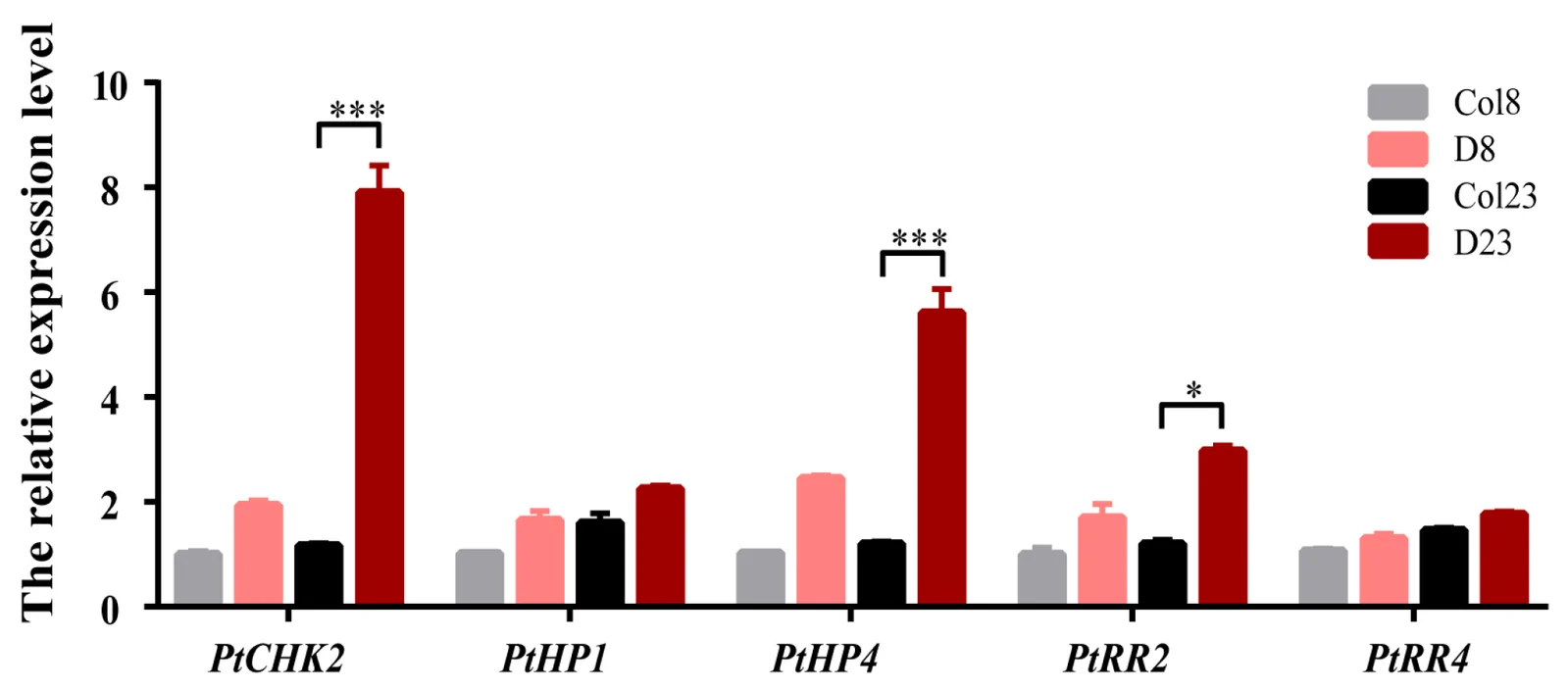
The qRT-PCR validation of *PtTCS* genes. Asterisks indicate significant differences: * *p* < 0.05, *** *p* < 0.001.

All the five *PtTCS* genes were used to perform qRT-PCR to validate the RNA-seq data. Among these genes, *PtCHK2*, *PtHP4* and *PtRR2* showed higher expression in long-term drought (23 days) and showed significant changes (Figure 8), and the other two genes were also induced to varying degrees. The validation results indicated that five *PtTCS* genes were induced by drought treatment and hence validated the RNA-seq data.

## 4. Discussion

Cytokinin signaling transduction pathway has been extensively characterized in many plant species (e.g., *A. thaliana* [47], *Oryza sativa* [48,49], and *Populus tomentosa* [50]). However, due to limited genomic resources, in conifers, no systematic study has been reported to date. Recently, with the advancements of next-generation sequencing technologies, the plant genomics landscape has been transformed, and large-scale sequencing information became available. The availability of *P. tabuliformis* genome assemblies opened new opportunities for more precise genome-wide studies [40]. Here, we present the first comprehensive genome-wide identification and characterization of cytokinin signaling components in a conifer.

A total of 22 *PtTCS* genes in *P. tabuliformis* genome were identified, (Table 1), which include three *PtCHK*, four *PtHP*, and 15 *PtRR* genes. This number is notably lower than those reported in angiosperms, such as 29 in *A. thaliana* [47], 41 in *Populus trichocarpa* [50], 40 in rice [48], and is comparable to the 17 members in the moss *Physcomitrella patens* [34]. The relatively small TCS complement in *P. tabuliformis* suggests that expansion of this gene family has been limited in gymnosperms, consistent with the view that the CK signaling pathway represents an evolutionarily ancient and core regulatory module that has remained relatively compact outside the angiosperm lineage. Phylogenetic analysis further supports this: the three PtCHK proteins clustered with CHKs from *P. patens* rather than with those from angiosperms (Figure 2), and the receptor copy number was relatively stable across species (two to four in most plants) [34]. The four authentic PtHPs grouped with angiosperm HPs, yet the presence of additional pseudo-HP genes in *P. tabuliformis* mirrors a pattern previously noted in gymnosperms [9] and may reflect lineage-specific evolutionary events related to environmental adaptation. Type-A RRs from angiosperms and the moss formed distinct clusters, while type-B RRs were broadly dispersed across the tree, implying a higher degree of evolutionary conservation in type-B RRs. Conserved domain analyses confirmed that the core domain architecture of PtTCS proteins is shared with other species; however, multiple sequence alignments revealed altered key amino acid residues in certain conserved domains (Figures S3, S5 and S6), which could potentially modulate protein function and deserves further investigation.

The expression patterns of *PtTCS* genes across organs were generally broad and without pronounced tissue specificity, reminiscent of the overlapping expression profiles reported for Arabidopsis TCS members [26,27]. This widespread expression is consistent with the pleiotropic roles of cytokinin in plant development. Nearly all genes were detected in roots, in agreement with the fact that roots are a major site of cytokinin biosynthesis [51]. A few genes showed organ-enriched expression, such as *PtRR6* in pollen and several *PtRR* genes in male and female cones, suggesting specialized contributions to reproductive development. The high root expression of *PtHP1*, *PtHP4*, and *PtRR4* hinted at potential involvement in stress responses, which was further explored.

Cis-element analysis of the promoter regions revealed an enrichment of elements associated with hormone response, light response, development, and abiotic stress, including drought-responsive MBS, low-temperature-responsive LTR, and anaerobic-responsive ARE elements (Figure 4). Consistently, GO analysis linked the PtTCS proteins to environmental stimulus responses (Figure 5). Together, these features support the notion that *PtTCS* genes may function in both developmental processes and adaptation to environmental fluctuations.

A central finding of this study is that the drought-induced expression changes of several *PtTCS* genes in *P. tabuliformis* contrast with the established paradigm in angiosperms. In Arabidopsis and other angiosperms, many TCS components act as negative regulators of drought tolerance, and their expression is frequently suppressed or functionally inhibitory under water deficit [19,26,27,29,52,53]. Correspondingly, drought generally reduces endogenous cytokinin levels in angiosperms [5,31,32]. In our study, five *PtTCS* genes (*PtCHK2*, *PtHP1*, *PtHP4*, *PtRR2*, and *PtRR4*) were significantly upregulated under drought, and rewatering reversed these changes (Figure 7). The cytokinin content in plants is regulated by cytokinin biosynthesis and cytokinin oxidase [51,54]. Moreover, we observed upregulation of two *PtIPT* genes and repression of six *PtCKX* genes under drought conditions (Figure S7), pointing toward enhanced cytokinin biosynthesis and suppressed degradation. These observations align with a recent report that active CK content increases in drought-stressed seedlings of *Pinus sylvestris* and *Picea abies* [35], and they collectively suggest a divergent drought response model in conifers: rather than dampening CK signaling, drought may elevate CK levels and promote signal transduction, leading to the activation of downstream response regulators. Based on these observations, we propose a working hypothesis in which drought-induced CK accumulation in *P. tabuliformis* activates signaling through *PtCHK2* and the phosphorelay via *PtHP1/PtHP4*, ultimately inducing *PtRR2* and *PtRR4* to regulate drought-responsive gene expression. Whether this reflects a distinct adaptive strategy related to the deep evolutionary divergence between angiosperms and gymnosperms remains an intriguing question.

While transcript level changes alone do not establish functional roles in drought tolerance, they provide a valuable basis for candidate gene identification. The differential expression observed here highlights several promising targets for further investigation. The comprehensive genomic and expression framework established in this study will facilitate future functional studies, including genetic manipulation and phenotypic analyses, to directly test the roles of these candidates in drought adaptation.

In conclusion, this work comprehensively identified genes related to the cytokinin signaling pathway in *P. tabuliformis* and reveals drought response patterns that differ markedly from the angiosperm model. The integration of genomic, phylogenetic, and expression data not only advances our understanding of CK pathway conservation and divergence but also highlights *P. tabuliformis* as a valuable system for studying alternative modes of hormone-mediated drought adaptation. Future studies combining functional genetics and metabolomics will be essential to test the proposed model and to determine how gymnosperms have rewired an ancient signaling pathway to cope with water limitation.

## 5. Conclusions

This study provides the first comprehensive genome-wide identification and characterization of the cytokinin signaling gene family in a conifer, *Pinus tabuliformis*. A total of 22 *PtTCS* genes were identified, comprising three receptor genes, four histidine phosphotransfer genes, and 15 response regulator genes. Systematic analyses of gene structure, conserved domains, phylogeny, chromosomal distribution, cis-regulatory elements, and expression profiles established a solid molecular framework for this core signaling pathway. Notably, the expression patterns of several *PtTCS* genes under drought stress, together with the expression shifts of cytokinin biosynthesis and catabolism genes, differ markedly from the canonical model in angiosperms, supporting a divergent drought response mechanism in conifers. These findings highlight *P. tabuliformis* as a valuable system for exploring alternative hormone-mediated drought adaptation strategies and lay a necessary foundation for future functional dissection of cytokinin signaling in gymnosperms.

## Figures and Tables

**Table 1:** *PtTCS* genes in *Pinus tabuliformis*.

Gene Name	Chromosome Locus	Amino Acid Length (aa)	GRAVY	pI Value	MW (Da)
*PtCHK1*	Pt6G32970	1031	−0.215	6.64	114,289.78
*PtCHK2*	Pt9G60240	1036	−0.145	6.05	114,135.28
*PtCHK3*	PtXG27360	1063	−0.188	7.82	116,726.51
*PtHP1*	PtXG46570	151	−0.485	5.00	17,527.75
*PtHP2*	PtQG24560	236	−0.026	5.69	27,220.45
*PtHP3*	PtQG24580	156	−0.308	5.13	18,143.7
*PtHP4*	Pt1G42860	151	−0.359	4.81	17,393.74
*PtRR1*	Pt8G13520	538	−0.501	6.86	59,678.52
*PtRR2*	Pt9G56450	548	−0.456	5.73	61,032.23
*PtRR3*	Pt9G55570	548	−0.430	5.64	61,108.43
*PtRR4*	PtQG11040	570	−0.460	5.51	62,880.16
*PtRR5*	Pt3G03540	756	−0.433	6.37	82,259.72
*PtRR6*	Pt4G04280	828	−0.502	5.83	89,739.45
*PtRR7*	Pt8G42410	483	−0.430	5.25	52,356.06
*PtRR8*	Pt5G19950	184	−0.482	6.17	20,533.31
*PtRR9*	Pt6G44810	200	−0.094	8.52	22,277.17
*PtRR10*	Pt3G60260	204	−0.427	5.55	22,645.88
*PtRR11*	PtJG44410	175	−0.603	5.77	19,578.07
*PtRR12*	Pt6G45400	196	−0.120	6.83	21,564.03
*PtRR13*	PtJG44210	156	−0.477	5.58	17,455.81
*PtRR14*	PtJG44180	159	−0.447	5.82	17,779.24
*PtRR15*	PtJG44170	159	−0.455	5.58	17,709.05

## Data Availability

The protein sequences of all genes analyzed in this study are provided in the Supplementary Materials. The transcriptomic data supporting the reported results are publicly available in the Chinese Pine Information Resource database and can be accessed via http://conifers.cn/CperProject/front/#/ (accessed on 28 September 2026) using the gene IDs provided in the article.

## Supplementary Materials

The following supporting information can be downloaded at https://www.mdpi.com/article/10.3390/metabo16100741/s1, Supplemental Table S1. All protein sequences of PtTCS. Supplemental Table S2. Conserved motifs and domains across the 22 PtTCS proteins. Supplemental Table S3. Full length protein sequences of *Physcomitrella patens*, *Populus trichocarpa* and *Arabidopsis thaliana*. Supplemental Table S4. Prediction of subcellular localization of PtTCS proteins. Supplemental Table S5. Cis-elements of *PtTCS* genes. Figure S1. The motif of PtCHK and PtHP proteins. Figure S2. The motif of PtRR proteins. Figure S3. Multiple sequence alignment of PtCHKs. Figure S4. Schematic representations for the chromosomal distribution of *PtTCS* genes. Figure S5. Multiple sequence alignment of PtHPs. Figure S6. Multiple sequence alignment of PtRRs. Figure S7. Expression patterns of *PtIPT* and *PtCKX* genes under the drought.
