# Supplementary material for: Genome-Wide Two-Component System Genes of Cytokinin Analysis and Their Functions of Drought Stress Response in Pinus tabuliformis
Source: Metabolites. 2026 Oct 1;16(10):741. doi: 10.3390/metabo16100741 (PMC13707277; doi:10.3390/metabo16100741)
Supplement: Supplementary file 1 [file metabolites-16-00741-s001.zip › Supplementary Table S1.pdf]

Protein sequences of PtTCSs

>PtCHK1
MSNSLGFSIPSKLPARFSQGCGLRLPSDMTCSLEAKMLTQGNGFHGGGVVSSYIYQSCGLS
RLALAVKWWGQSTVSNTKKQKPSKWPKLETKWHTKLFVLWVLVAAIVSMWIFYSMDY
DIIERRKETLASMCDERARMLQDQFSVSMNHVHALAILVSTFHHRKHPSAIDQKTFAEYT
ARTAFERPLMSGVAYAQRVHHSEREQFEKWQGWTIKSMKSREPSPIQDEYAPAIFFQETISY
VGSLDMLSGEEDHENIVRARATGKGVLTSPFRLLESNHLGVVLTFPVYYTDLPPDATPEER
NRATAGYLGGAFDVESLVENLLRQLAGNRAIIVNVYDITNSSTPLIMYGPQVTDTVVPHVS
HLDFGDPFRRHVMHCRFNEKPAIPWSAITTSLGIFVIAVLVGHIIYAAVNRIAKVEEDFRKM
EELKKQAEAADIAKSQFLATVSHEIRTPMNGVLGMLQMLMDTDLDATQQDYARTAQASG
KALIALINEVLDQAKIESGKLELEEVPFDLRSILDDVLSLFSGKSRDKGIEFTERGHICVSVH
LAENVKAVREANVEAFSKQLTEDRKQSSKSSCNTLSGFEAADGTNSWETFRLLLSDGIMN
QQPGTINKLSFAETSDTVNLVVSVEDTGVGIPIHAQRRVFMRFMQADSSTSRTYGGTGIGL
SISKRLVELMSGEMSFESKPAVGSTFTFTVVLKKAHTSQDMERHQPEPLPTIFKGMRAVVV
DGRPVRAEVTKYHLRRLGVQVEIANDQKSALAIISGGENGDIRCKATGNTDMVLVDKEA
WGTGTGLTFPQLLRESRLSSKAGSCPMLPRMILLATSINSVDSEKAKAIGFVETVIMKPLRA
SLIAACLQQALGIGDKRQQGKGVMDGSPSLHSLLLGKNILVVDDNKVNRRVAVAALKKY
GAKVENADSGKDAIAMLQPPHKFDACFMDVQMPEMDGFEATRQVRNIEQQINEHIESGV
ASREAYGDVDRWHVPILAMTADVIQATNEECTRCGMDGYVSKPLEEEQLYREVVRFLDT
KTKPT

>PtCHK2
MRMCRAEWKKIHQAQEQESKEESSKLLAGGWRGGTHGGHRTTGNGGTGVIHGLLSWQS
IDGFLSERVGERWNTMAVIVWVLLGSSVAIYSFYSMTRETSRRRQETLANMCDERARMLQ
DQFVASMNHVQALTVLVSTFHLEMKPSALYQDVFAAYTARTSFERPLMSGVAYAPRVLHR
DREEFERQQGWSIKHMNTKETQAPQEEYAPTVFSQETVSYLISLDMMSGQHDRENILRAR
ETGKGALTTPFRLVSSHLGVVFTFAVYNRGLPPDATPQQRTEATAGYLGGAFDVESLVENL
LHQLAGNFPINVNVYDTTNESFPVLMYGTGSNGYHISELDFGDPDRKHELHCGFSDDPKL
PYTAIRTSSGIFVIIILVGHILFAAISRIKKVEEDCREMEELKGRAEAADVAKSQFLATVSHEI
RTPMNGVLGMLQMLMDTDLDATQKDYAQTAQASGKALITLINEVLDQAKIESGRLELET
VPFHIRTVLDSVLSLFSAKTQAKGIELAVFVSERVPEIVIGDPGRFQQIITNLVSNSVKFTEN
GHIFVCVHLAQEIDVLTHRLSAYKKIGEDPAINNYSLSNTLSGSEAADTRNNWESFKILHEI
GSDELFSESDGRGRIARLTDVVKLAVSVEDTGIGIPLPAQHRVFTPFMQADSSTSRTYGGTG
IGLSISRCLIELMGGEIRFISRPGIGSTFSFTALFKVGQAGADGDGDLLRGARLPTHFKGMK
ALVLDGNPVRSLVTKYHLQRFGIEVDSITSSKVALSMLNGMDGFPTEGCSVKDGIDMVLIE
KDAWGPGTGILFPSQVRVGLFPRGPFLQSKGLLKMILLATSLTAEETQKAKAAGFAETVIL
KPLRASMLAVCLQLALGFCNRREHLREPLKTSSPLSNVLSGKSILVVDDNIVNRRVAAGAL
KKYGANVICTDSGKSAISMLRQPHNFNACFMDVQMPEMDGFEATRQIRAAELANMECTS
NGGETLATNNRWHVPILAMTADVIQATHEECLRCGMDGYVSKPFEEEQLYKALAPFFEEP
LVR

>PtCHK3
MSLASILSTLSSAIGMYQACGWAWHNSNSTMNCSRIAQNTGLNAGLLDGSVNRSINHHPP
GAGTGRLTPSWSGRSPNSTNSKKQNPTEGPKGGGTWRWKLLILWVLGGFVGSLWAFYCM
NTAMVERRKDLLTSMCDERARMLQDQFAVSMNHVHALALLVSTFHIGKQPSAIDQKTFA
EYAKRTAFERPLTSGVSYAQKVLHSQREEFERQQGWEIKMMKTKEISPVQDEYAPVIFSQD

TVSYIRSLDMMSGKEDRENVLRARASGKGVLTSPFTLESNHLGVVLSFTVYKTDLPTDAT
PEERIEATAGYLGGSFDVESLVENLLQQLAGKHAITVNVFDITNSSKPVIMYGPPVTNNGLS
HVSALDFGDPFRKHQMLCRFTQEPPLPWSAITTSLGILVIALLVGHIFYAALNRIAKVEEDF
RKMAELKLKAEAADIAKSQFLATVSHEIRTPMNGVLGMLQMLMDTGLDATQQDFARTAQ
ASGKALITLINEVLDQAKIESGRFELEAVPFDLRAILDDVLSLFSGKSRNKQIELAVYISDKV
PETVIGDPGRFRQIVINLVGNSIKFTEHGHIFVSVHLVEELQSVEVNSETSKRFTEVKNGSSS
SCNTLSGCEAFDVRNNWENFKLLHSNGTYKLPNTLKDCSSPETSQTVNLCVSIEDTGVGIP
LNAQERVFVPFMQADSSTSRTYGGTGIGLSISRFLVELMNGRMDFVSKPAIGSTFVFTAVFK
KGHTCSQLQDMKLRQSEALTTVFKGMRALVVDGRPIRASVTEYHLKRLGVQVEIVTNLK
SAIAAVGGGRNGDIRSSEARNINMILVDKDAWGQGSGLTFPKLLKELRQNGRTRYSSELPK
MILLATSIGDDKTDTAKAAGFVETVIMKPLRASMVAACLQQALGVGNKRKQGKGSPPPL
QSLLFGKQILVVDDNKVNLRVAAGALKKYGANVECAESGKSAVEMLKPPHKFDACFMD
VQMPEMDGFEATRQVRDLERTVNEQIDSGAISKEIDGNISKWHVPILAMTADVIQATHEEC
SKCGMDGYVSKPFEEEQLYRAVAEFFESKLTSTA
>PtHP4
MAVSQMQQRYINFTNSLFQEGFVDEQFTQLQQLQDESNPDFVAEVVFLFFEDSDKLLADL
SKTLNQEPVDFKRVDGCVHQFKGSSSSIGARRVMNTCVTFRAFCQDKNREGCLYCLQQM
KQECYLVKNKLETLFQLEKQIVEAGGTVPMSE
>PtHP2
MPLISDANGILCQFWPKCEGISRLACAATIFFKHKKKKHLYSVTSEPCLFVLDFFYINPTTA
RVQSIGGLSLLIGSVLWDSATIIYLLFTVGNYKSVATGLPVWMLRFGMDVIRLKQRLIQLTN
SLFQEGFLDEQFTQLQQLQDENDPEFVTEVISLFFEDSEKQLHNLANELDNEAIDYKRIDA
YVHQFKGSSSRCLQCLQQLREEYYLFKNKLQHLFHLEQQILSAGGTLPMME
>PtHP3
MFSFRMDVIRLQQRLIQLTNSLFQEGFLDEQFTQLQQLQDDSNPDFVTEVVVLFFEDSEKL
LHNLTNALNKEVIDYKKMDAHVHQFKGSSSSVGAQRVKNLCIALRTFCNDNNRAGCIQC
LQQLKDEYYLFKDKLQDLFHLEQQILSAGGTLPMME
>PtHP1
MDVVQLQRRFIDYTSSLYREGFLDEQFTQLQQLQDESNPEFVAEVVSLFFEDAEKLLADLS
KTLAQQPIDFKKVDAHVHQFKGSSSSIGAHRVKNECISFRAFCEQRNREGCLQCLEQLKQ
EYYNVKNKLETLFQLEHQILEAGGTIPMSE
>PtRR1
MSPLVDRDPQVAKGEAFPVGRSCSDSCCHVGGPTKAAAVTSDEEKFSPSGLQVLVVDDDT
FCLAVLERMLRQCEYNVTVCTRVSQAISLVKENREGFDIVMSDVYLPGEDGFKLLEVVGI
GLGLPVIMMSANGETNVVMRSIRGGACDYLIKPIRTEELRTIWQHVVRRHRQQCHRHDG
KDNHGDFSDHGGVLGEFKKRKHSSCQDIKDLNSLKTARVHWTAQLHQRFVKAVNQTGL
DTPKRILEIMNVPGLTRENVASHLQKYRLYLKRLSGSIPEPRPVASFQAARDGKFGGTMQI
RKRGKPTPSSSSSSSSSSAIKTSPLSLELSRNLDHATLKSLEQFQAYERKLVANRVQMLRGA
ATAAAPSSYMRTNVHVDQGYENGGELRIITHAGRLRKNLQGGGGHRACPADESAAQSFK
VDFANLAETKPLTKSFGLSTHQPQFQNPAPTTETPEENDVHKEEAKRLIDETQTIDQEELL
FNNFVEENFPHMKIPSDHDQMPTLIASAGDLSEEYFMLGEAFLPRLGDKEEITQVHIP
>PtRR2
MMMDDKSCVGKLEDECRSIMQIGNFSPIGLKVLVVDDDPLCLLVLERMLRQCNYNVTTC
SRVPQAISMVKENRDRFDLVMSEVYLPDEDGFRLLEIVGLGLDLPVIMMSTNGDTSVVMK

GITDGAYDYFIKPIRLEELRNIWQHVVHIRGRESLKDDLGECEDCEISDSPDTSSKKRKDTS
SGDFSDEVIDDISSLKRARVHWTVQLHQQFVIAVNQLGIEKAVPKKIVEIMKVQGLSRE
NVASHLQKYRLYLKRLSGAISEPHPVASFQAADDSISGGMMKLKQGEKGVASSSGAKGLN
LGTGVSSSLGVRGLDPSTLKSLQQYRAYQQKLVANRAQIFGGIGVRPPKYSDTAKVPESM
GGSKSGLQRMSSVDMGLLWKAQREQTADENEKQGIANLQNLPQKRSNSSEQRRFRGSHP
KHSRTRTMTDLPMIFQSAKPEVTFQKTPPAKSMLQDVDELQLMEDISWLDNTFKPESPRA
AVISFDSFVQDFEGSSLSAATIEESNRHAFASTDFEGQRKAGVENEEFFSSGSTDISDYLVDD
LMPHPR
>PtRR3
MMMDDKSCVGKLEDECRSIMQIGNFSPIGLKVLVVDDDPLCLLVLERMLRQCNYNVTTC
SRVPQAISMVKENRDRFDLVMSEVYLPDEDGFRLLEIVGLGLDLPVIMMSTNGDTSVVMK
GITDGAYDYFIKPIRLEEIRNIWQHVVHIWGRESLKVDLGECEDCEISDSPDTSSKKRKDTS
GGDFFDEVIDDISSLKRARVHWTMQLLQQFVSTVDQLGIDRAIPKKIIKIMKFEGLSLENVA
SHLQKYRLHLKRLSGAISEPHPVASFQAADDSISGGMMKLKQGEKGVASSSGAKGLNLGT
GVSSSLGVRGLDPSTLKSLQQYRAYQQKLVANRAQIFGGIGVRPPKYSDTAKVPESMGGS
KSGLQRMSSVDMGLLWKAQREQTADENEKQGIANLQNLPQKRSNSSEQRRFRGSHPKHS
RTRTMTDLPMIFQSAKPEVTFQKTPPAKSMLQDVDELQPMEDISWLDNTFKPESPRAAVIS
FDSFVQDFEGSSLSAATIEESNRHAFASTDFEGQRKAAVENEEFFSSGSTDISDYLVDDLMP
HPR
>PtRR4
MRPRVRLAEESRCMKQIENFSPRGMEVLVVDDDPLCLMVLERMLRQCDYNVTTCSRVSE
AISMVRERKDRFDLVMSDVYMPNEDGFKLLEFIGLTLDLPVIMMSANGETSMVMKGITH
GACDYLVKPVRMEELRNIWQHVVRRRGREYLREESGDSENETSKLVESSESSSKKRKDSQ
NLDHSSEIIDDISSLKKTRIHWTVQLHQQFVAAVNQLGIDKAVPKRIIEIMGVHGLTRENVA
SHLQKYRLYLKRLSGSIPAPYPVASFQAAEGGKSGGRMHIQPPAKSGASSPVLTKGLNLGF
ATSPNSTGSFGIGGIDQGTLKSLEQYRAYEQKRAANRAQVLGGIGIMPSKQSSVGQLPAES
KGGLQRMASVDLSLLWKAQRDQASSQKPAGLNVGDDLADQSFKKTVKLENVQLEPFPV
QPASKHSRAHSMSNVLTDFCNIDPPLTGFQRSGMLKEINELQPMEGISWEGSTSAKGEFKP
VEEMSWKGSTSTGGAGAEVGVDTSVDLFDDFVEEYGITSLSPPPTEMDEQSFAGADFEG
MGEAGNQDPSPITPDDIADYLFDDFLPRSR
>PtRR5
MNSISEMTTMQSLQRSSMSTASSFPSESSGGFDEKSGFNQKSRFPDRFPAGLRVLVVDDDPI
CLKIIEKMLRRCLYEVTTCSHATVALSMLRERKGSFDLVISDVYMPDMDGFKLLEHVGLE
MDLPVIMMSADGETSVVMKGIKHGACDYLLKPIRIEELKNIWQHVIRKKRNLGRELEYSG
SAEDSDKNKKGSDDIEYASSVNEGTESNSKTSKRRKETKEEDDDFEQENDDPMSLKKPRV
VWSVELHQQFVNSVNQLGIDKAVPKRILEMMNVHGLTRENVASHLQKYRLYLRRLSEVA
QQQSGLNSSFGGTAEVNLGSMCALGRLDLQAFAASGQKSPQTLVAMQAELMGKINAKNG
LGISGDTFCLPTAFHGVSSNSLNSLGFGQQLMNNQGGVLQGFPSGLKLKQLSLSQSQPNIS
SYENGVFSMNDTSPGLPFLPQLSTAAVALRDLDHIYGSNNNLGLDLDDKASMLKLLQQQ
QLHQTPGPLPHQSGAQLHGQQVLNLPSSSLLGQRTLLNTLSTLSNANSNQGNSLIGPGSAQ
KNLITPEGHDLTLNACNAYFGIRRMEDVNQSLNSMPVNSVFQPRTVASTPLSSGAEGVMS
LPGMNIPRETPEVFNSQALRSSGVNCNALNGFAPGLTQGNVLGCQVPKAAFNQGLGPMVI
PTLNPHYGQTSSGLQGLNPGIQDHVLSQSLGFSGKGSNLPNRFTSNDGHLRTISNTPRQSD
QSRADTGTRLKDRVSDLMEGTRLAEREFSLDGYN

>PtRR6
MATGPAFECSQGLPEFAKKESCSSVNPVNSVSVTSPVMEGVPDQFPAGLRVLVVDDDPTC
LKILEKMLQTCRYEVTTCSRATVALSMLRERRGAFDLVISDVYMPDMDGFKLLEHVGLE
MDLPVIMMSADGETSVVMKGIKHGACDYLLKPVRIEALKNIWQHVIRKKRNESKELEHS
GSVDDSDRHKKGSDDVEYASSVNEGNNGMWKPSKKRKEAREDEDDGEQDNDDSSNLK
KPRVVWSVELHQQFVCAVNQLGIDTVPKRILELMNVQGLTRENVASHLQKYRLYLRRLSG
VGQQQSGFNSSFGGSMEANFGSISSLDRSDLQALAASGQISRQTLAAFQGGLLGRVNGND
VGMSGVDPTFLLQPDLQGLNCSLTDRARFGQPLLNSQRNLLQGLPTGLELKQLSQSHQH
MPSFGNLGMPMDDSSPGFPIVQQQLPTASIELGGMGQISSINNNTALNPHNNSLMVQMMQ
QQQQHPLNHHQQQMPTSAGQQQHPLNHHQQQMPAGSRQQQQSGEQNQNEQLEGVQVL
NMPSPNLLQQQMLSNDIGSIANPLSTVGNSSTLLGNTTVGTSVSGQANQIPTGIHGLTLPQS
NASVGNGRTPTVDYQNHYHPIMQGNNYSLARAVGTTTPLASVGTCGDMSMAGVGNLGG
MSEIVNSHTLRSSNTNFSTLNGFGQNLSQSSKQGWHGQSLAQNSVLSQTGNLVPNSRYSQ
PFPASQSMNISSTQNKGQVGGFVTCGKGLGLPSRLSTDAGDTRTGTRLQREQSADSALRL
KEDGLSSVMPIAKFEGGLLNDHYMQEDLMSVLLKQQQEGVGLAESEFSTDGYQLDVK
>PtRR7
MGRSEGGLAVVTMQQPPHANTGGVPGNMSMKDGKSNDKVKVMNDEEANDDFPIGMRV
LVVDDDPICLLLLESLLRRCKYNVTSCGQAITALNLLRENKDKFDLVISDVYMPDMDGFK
LLELVGLEMDLPVIMMSANGETSAVMKGITHGACDYLLKPVRLEELKNIWQHVVRKRRT
ESKDRDNLDHGDANDKFNHGPEDGEHGSSANDGTDRTWKLNRKRKEQNDDEDDDDDE
HDMEDPSTSKKPRVVWSVELHQQFVNAVNSLGIDKAVPKRILELMNVQGLTRENVASHL
QKYRLYLKRLSGVASQQGSMGNAFGGGRESSFASLCQVDGIGDLQVLAQSGQLSARALE
SLQAGVLGRLNGSVGLGLPGLNPSGMLQFASLPGLGSNNSIGRAQGIATVNNPGTAFPCLS
TGIELDQLQQKQQITRLGDISSPVDDPAGFRTMQRQLTVTNSLPVGLGGGSSGNIPVNSTN
NALVLQLMQQ
>PtRR8
MARNESGDAASTSEFHVLAVDDSVLDRKLIEKLLKISSYRVTTVDSVRKALQFLGLEDDE
DDLNVDNIQVNMIITDYCMPGMTGYDLLRRVKESSAVKDIPVVIMSSENVESRISRCMSEG
AEEFLLKPVQLSDVKKLRSHILNKGRSRDQQSTSSSNNKRKTMTDSLSPPAERRPRPSGLT
VA
>PtRR9
MSVSSAVGRMGGKVCEGVFEEAVAFAGCETHVLAVDDCVIDRKIIERLLETDTVKVTTVD
SPTRALEMLGLSQEPATTPPCKNVFKFSMIITDYCMPEMTGYDLLKKLKETKGLKEIPVVII
SSENVPQIIQRCLDEGAMDFIIKPVQLEDVNKIRRHIIQLQSMHAPLTTVSRKRKALPTNLA
GEASERRPRIKRVRVP
>PtRR10
MGRDGGVLLQRVLEAESGSSSSEGSERPVASSQFHVLAVDDSIVDRKVIERLLKISAYKVT
TVDSGRRALEFLGLDEDHSSVDLDNLNVNMIITDYCMPGMTGYDLLKRIKESKKLKEIPV
VIMSSENVVPRINRCLEEGAEEFILKPVKLADVERFKGHIIKGLSKEQQQPASNKRKSMSE
DLDDLPAQPPERRPRLGELSGA
>PtRR11
MGRKGSGDSQLRTEDSMPSTSDQSSRQGESTSQLHVLAVDDSTIDRKVIEKLLKNSCYRV
TTVDSGRRALDFLGLGDDHNAVNDLQVNMIITDYCMPGISGYDLLKRVKESSKLKEIPVV
VMSSENVPSRKRRCLDEGAEEFILKPVQISDVKRLREYMSRVQEESSYCPNGSLN

>PtRR12
MEEDGVKITFGDSQAITLGEAATDGHEMHVLAVDDCRVDRMVVERLLRSDSVKVTAVDS
AAKALELLGVNKEHATATVCTNPLKFSLIITDYCMPELTGYDLLKRLKEIKGLKEIPVIIMSS
ENVPQRIQRCLDEGAEDFIVKPVQRDDIKKITGYIKVSKPVATTPICRKRKVSSVVLAGDNS
ERRPRISGVTVA
>PtRR13
MSSTSDHSVEEGESTSQVHVLAVDDSVIDRKVIEKLLKNSCYKVTTMDSGRRALEFLGLG
DDQNVANVNMIITDYCMPGMSGHDLLKRVKESSKLKEIPVVVMSSDNESSRKRRCLDEG
AEEFILKPVQISDVKRLRDYICRDHKSDSYCPTGPSI
>PtRR14
MSSTSDHSIEEGESTSQIHVLAVDDSVIDRKVIEKLLKNSCYKVTTMDSGRRALEFLGLGD
NQNVANGLQVNMIITDYCMPGMSGHDLLKRIKESSKLKEIPVVVMSSDNESSRKRRCLEE
GAEEFILKPVQISDVKRLRDYICRDHKSDSYCPAGPSI
>PtRR15
MSSTSDHSSQEGESASQLHVLAVDDSVIDRKVIEKLLKNSCYKVTTMDSGRRALEFLGLG
DDQNVANGLQVNMIITDYCMPGMSGHDLLKRVKESSKLKEIPVVVMSSENESSRKRRCL
DEGAEEFLLKPVQISDVNRLRDYIRIDHKSDSYCPTGASI
