# Supplementary material for: Genome-Wide Two-Component System Genes of Cytokinin Analysis and Their Functions of Drought Stress Response in Pinus tabuliformis
Source: Metabolites. 2026 Oct 1;16(10):741. doi: 10.3390/metabo16100741 (PMC13707277; doi:10.3390/metabo16100741)
Supplement: Supplementary file 1 [file metabolites-16-00741-s001.zip › Supplementary Table S2.pdf]

>AT5G35750_AHK2
MSITCELLNLTSKKAKKSSSSDKKWLKKPLFFLILCGSLVIVLVMFLRLGRSQKEETDSCNGEEKVLYRH
QNVTRSEIHDLVSLFSDSDQVTSFECHKESSPGMWTNYGITCSLSVRSDKQETRGLPWNLGLGHSISSTS
CMCGNLEPILQQPENLEEENHEEGLEQGLSSYLRNAWWCLILGVLVCHKIYVSHSKARGERKEKVHLQEA
LAPKKQQQRAQTSSRGAGRWRKNILLLGILGGVSFSVWWFWDTNEEIIMKRRETLANMCDERARVLQDQF
NVSLNHVHALSILVSTFHHGKIPSAIDQRTFEEYTERTNFERPLTSGVAYALKVPHSEREKFEKEHGWAI
KKMETEDQTVVQDCVPENFDPAPIQDEYAPVIFAQETVSHIVSVDMMSGEEDRENILRARASGKGVLTSP
FKLLKSNHLGVVLTFAVYDTSLPPDATEEQRVEATIGYLGASYDMPSLVEKLLHQLASKQTIAVDVYDTT
NTSGLIKMYGSEIGDISEQHISSLDFGDPSRNHEMHCRFKHKLPIPWTAITPSILVLVITFLVGYILYEA
INRIATVEEDCQKMRELKARAEAADIAKSQFLATVSHEIRTPMNGVLGMLKMLMDTDLDAKQMDYAQTAH
GSGKDLTSLINEVLDQAKIESGRLELENVPFDMRFILDNVSSLLSGKANEKGIELAVYVSSQVPDVVVGD
PSRFRQIITNLVGNSIKFTQERGHIFISVHLADEVKEPLTIEDAVLKQRLALGCSESGETVSGFPAVNAW
GSWKNFKTCYSTESQNSDQIKLLVTVEDTGVGIPVDAQGRIFTPFMQADSSTSRTYGGTGIGLSISKRLV
ELMQGEMGFVSEPGIGSTFSFTGVFGKAETNTSITKLERFDLAIQEFTGLRALVIDNRNIRAEVTRYELR
RLGISADIVSSLRMACTCCISKLENLAMILIDKDAWNKEEFSVLDELFTRSKVTFTRVPKIFLLATSATL
TERSEMKSTGLIDEVVIKPLRMSVLICCLQETLVNGKKRQPNRQRRNLGHLLREKQILVVDDNLVNRRVA
EGALKKYGAIVTCVESGKAALAMLKPPHNFDACFMDLQMPEMDGFEATRRVRELEREINKKIASGEVSAE
MFCKFSSWHVPILAMTADVIQATHEECMKCGMDGYVSKPFEEEVLYTAVARFFEPC
>AT1G27320_AHK3
MSLFHVLGFGVKIGHLFWMLCCWFVSWFVDNGIEDKSGLLVGSVGDLEKTKMTTLKKKNKMWFWNKISSS
GLKIPSFSYQFLGSVKFNKAWWRKLVVVWVVFWVLVSIWTFWYFSSQAMEKRKETLASMCDERARMLQDQ
FNVSMNHVQAMSILISTFHHGKIPSAIDQRTFSEYTDRTSFERPLTSGVAYAMRVLHSEREEFERQQGWT
IRKMYSLEQNPVHKDDYDLEALEPSPVQEEYAPVIFAQDTVSHVVSLDMLSGKEDRENVLRARSSGKGVL
TAPFPLIKTNRLGVILTFAVYKRDLPSNATPKERIEATNGYLGGVFDIESLVENLLQQLASKQTILVNVY
DITNHSQPISMYGTNVSADGLERVSPLIFGDPLRKHEMRCRFKQKPPWPVLSMVTSFGILVIALLVAHII
HATVSRIHKVEEDCDKMKQLKKKAEAADVAKSQFLATVSHEIRTPMNGVLGMLHMLMDTELDVTQQDYVR
TAQASGKALVSLINEVLDQAKIESGKLELEEVRFDLRGILDDVLSLFSSKSQQKGVELAVYISDRVPDML
IGDPGRFRQILTNLMGNSIKFTEKGHIFVTVHLVDELFESIDGETASSPESTLSGLPVADRQRSWENFKA
FSSNGHRSFEPSPPDINLIVSVEDTGVGIPVEAQSRIFTPFMQVGPSISRTHGGTGIGLSISKCLVGLMK
GEIGFSSTPKVGSTFTFTAVFSNGMQPAERKNDNNQPIFSEFRGMKAVVVDHRPARAKVSWYHFQRLGIR
VEVVPRVEQALHYLKIGTTTVNMILIEQEIWNREADDFIKKLQKDPLFLSPKLILLANSVESSISEALCT
GIDPPIVIVKPLRASMLAATLQRGLGIGIREPPQHKGPPALILRNLLLGRKILIVDDNNVNLRVAAGALK
KYGADVVCAESGIKAISLLKPPHEFDACFMDIQMPEMDGFEATRRIRDMEEEMNKRIKNGEALIVENGNK
TSWHLPVLAMTADVIQATHEECLKCGMDGYVSKPFEAEQLYREVSRFFNSPSDTES
>AT2G01830_AHK4
MRRDFVYNNNAMFNPLTTHYSSDMNWALNNHQEEEEEPRRIEISDSESLENLKSSDFYQLGGGGALNSSE
KPRKIDFWRSGLMGFAKMQQQQQLQHSVAVKMNNNNNNDLMGNKKGSTFIQEHRALLPKALILWIIIVGF
ISSGIYQWMDDANKIRREEVLVSMCDQRARMLQDQFSVSVNHVHALAILVSTFHYHKNPSAIDQETFAEY
TARTAFERPLLSGVAYAEKVVNFEREMFERQHNWVIKTMDRGEPSPVRDEYAPVIFSQDSVSYLESLDMM
SGEEDRENILRARETGKAVLTSPFRLLETHHLGVVLTFPVYKSSLPENPTVEERIAATAGYLGGAFDVES
LVENLLGQLAGNQAIVVHVYDITNASDPLVMYGNQDEEADRSLSHESKLDFGDPFRKHKMICRYHQKAPI
PLNVLTTVPLFFAIGFLVGYILYGAAMHIVKVEDDFHEMQELKVRAEAADVAKSQFLATVSHEIRTPMNG
ILGMLAMLLDTELSSTQRDYAQTAQVCGKALIALINEVLDRAKIEAGKLELESVPFDIRSILDDVLSLFS
EESRNKSIELAVFVSDKVPEIVKGDSGRFRQIIINLVGNSVKFTEKGHIFVKVHLAEQSKDESEPKNALN

GGVSEEMIVVSKQSSYNTLSGYEAADGRNSWDSFKHLVSEEQSLSEFDISSNVRLMVSIEDTGIGIPLVA
QGRVFMPFMQADSSTSRNYGGTGIGLSISKCLVELMRGQINFISRPHIGSTFWFTAVLEKCDKCSAINHM
KKPNVEHLPSTFKGMKAIVVDAKPVRAAVTRYHMKRLGINVDVVTSLKTAVVAAAAFERNGSPLPTKPQL
DMILVEKDSWISTEDNDSEIRLLNSRTNGNVHHKSPKLALFATNITNSEFDRAKSAGFADTVIMKPLRAS
MIGACLQQVLELRKTRQQHPEGSSPATLKSLLTGKKILVVDDNIVNRRVAAGALKKFGAEVVCAESGQVA
LGLLQIPHTFDACFMDIQMPQMDGFEATRQIRMMEKETKEKTNLEWHLPILAMTADVIHATYEECLKSGM
DGYVSKPFEEENLYKSVAKSFKPNPISPSS
>Pp1s50_141V6.1
MHRATGSGRLGVKWWKDSGITCIKPKQKDSEAGSRYGHNWHTIAFIMWVVFMGAVSVSVYFAMRFDMLER
RRENLANMCEGRARMLEEQFKASMNHVRALTALLTTFHLGKQPSAINQETFATYAERTAFERPLMSGVAYAER
VLHANRNLFESKHGWTIKEMFSKEKQRDFDEYAPTTMTQETLSHLTSLDMMSGQEDRENIIRARESGKGALTN
PFRLLESKHLGVVFTFAVYTTDLPEDATKEDRIKATAGYLGGAFDVETLVENLLRQMAGSQIIAVNVYDITNETMP
LVMYGYTNVEKMQSTYVSDLDFGDPARRHEMRCRFKEDALPPWTAISTSIGIFVIAFLVGHMLHASRNRIDKVE
ENCRIFQELKMRADDAVIAKSQFLATVSHEIRTPMNGVLGMLQMLIDTPLNPAQLDYAQTALASGKALINLINE
VLDQAKIESGRLEFEHVPFDLRAILDDVLSLFHGKSRASGIELAVYVSARVPKILVGDPGRFRQIITNLVGNSIKFTE
AGHIFVCVHLNEDVNMVMDGSGGDADDTMDGEVESAGPLNDMNYRFNTLSGRETADYRNSWEMFERLLA
QEQSHNDTWSYSDSSDVVNLVVSVEDTGVGIPLHVQDRVFTPFMQADSSTSRNYGGTGIGLSISRCLVELMSG
EMGFVSRPKVGSTFTFTVELQRVPANIPERILREGRNSYLQTPPLTTCFQGMRVLVVDGRKVRCEVTRHHLKRL
GLQVEVAHTVSEAAEILSTVADQGTCSSRGSRGSLAGNIEMIFVDKDAWGPGTGMNFRSLLGESAIGHLGYQSS
RTSYQYRTSGRIPFGYPVIQSMKRMVLVATSMTDMELGMVKASGFVDTVVRKPLRASLIAACLKQILGVGDRSR
DQEQELRSGVTSLQTLLYGKRILVVDDNPVNRKVAAGALHKYGAQVECVYSGRAAIQKLKPPHNFDACFMDVQ
MPEMDGFEATGLIRKAEAAAAERGQAAGLPTFKRRLHVPILAMTADVIQATSEECMRCGMDGYVAKPFDEH
QLYQAVAKHFFDVN
>Pp1s194_72V6.1
MFYLRVKSVTRQEAKTSLSRVLKIFRFKKDLMSTQNGAKRGSYSSPCISNGSSRSKVPLKGGRGVYMHRTTGPG
RLGVNWWKDTGISYIRPTYKDSEARSRYGHNWHTKAFVVWVVFMGAVSLSVFLAMRFDMLERRRENLANM
CEGRARMLEEQFKASMNHVRALTALLTTFHLGKQPSAINQETFATYAERTAFERPLMSGVAFAERVLHANRKLF
ESAHGWTIKEMYSKDLQQDFDEYAPTTMSQKSLSYLTSLDMMSGQEDRENIIRARELGKGALTKPFRLLESKHL
GVVFTFAVYCTDLHDDAKKEDRIKATAGYLGGAFDVETLVENLLRQMAGSQIIAVNVYDITNETMPLVMYGHT
NVEKSKNTYVSSLDFGDPARRHEMRCRFKEDALPPWTAISTSIGIFVIAFLVGHMLHASRNRIDKVEENCRIFQEL
KMRADDAVIAKSQFLATVSHEIRTPMNGVLGMLNMLTNTRLNPAQLDYARTALASGKALINLINEVLDQAKIES
GRVELEHVPFDLRAILDDVLSLFHGQSRDSGIELAVYVSAKVPKILVGDPGRFRQIITNLVGNSIKFTEAGHIFVCV
HLNEDVNNLMDGDEGEADDTLDGEVESAGPLTDMNYRFNTLSGRETADYRNSWEMFERLLAQEKSHRSGVD
QDTRNYCDLNGAVNLVISVEDTGVGIPLHVQDRVFMPFMQADSSTSRNYGGTGIGLSISRSLVDLMSGEMEFV
SHPKVGSTFTFTAELQRVPANVPEHILREGRNCYLQTPPLTTCFQGMRVLVVDGRKVRCEVTRHHLQRLGLRVE
VAHTPGEAAELLTTTVDRGNRGSRDSQRSIVLLSWIEDIELIFVDKDAWGPGTWVNFRSLLTESFIGQLSHNQSG
LTSYQYRSAAGRLPNGYPTLNSMKKMVLVATSMNEMELSVVKASGFVDTVVRKPLRASLVAASLEEILGVGDG
GRDQGQNLQGGINSLQNLLTGKRILVVDDNPVNRKVAAGALNKYGAQVDCVNSGRAAIQSLKPPHNYDACF
MDVQMPEMDGFEATRLIRKAEAAAVDNWQAGRFSSFGRRLHVPILAMTADVIQATSEECMRCGMDGYVAK
PFDEHHLYQAVAKHFFGPN
>Pp1s252_49V6.1
MRQRKQLINPDSNQMSNQSGARSGVRSEARRGSESSPCISNGSSRSKVPLKCGSGVYMHRTTGPGRLRVKW
WNDSGNTYVRPTYNDSKAGPRYGHNWHTKVFVFWVVFMGAVSLSVYFAMRFDMLERRRENLANMCEGR
ARMLEDQFKASMNHVRALTALLTTFHLGKQPSAINQETFATYAARTAFERPLMSGVAYAERVLHANRAMFELTH

GWTIKEMFSKERQQDFDEYAPTTMSQETVFYLTSLDMMSGQEDRENIIRARESGKGALTNPFRLLESKHLGVV
FTFAVYSTDLPFDATKEDRIKATAGYLGGAFDVETLVENLLRQMAGSQIIAVNVYDITNDTMPLVMYGHTNVERS
DNTYVSLLDFGDPLRNHEMRCRFKEDALPPWTAISTSIGIFVIAFLVGHMLHASRNRIDKVEENCRIFQELKMRA
DDAVIAKSQFLATVSHEIRTPMNGVLGMLQMLMDTPLNSTQLDYARTAQASGKALITLINEVLDQAKIESGRLE
LEHVPFDLRAILDDVLSLFHGKSRASGIELAVYVSEKVPKILIGDPGRFRQIIINLVGNSIKFTEAGHIFVCVHLNED
VNMVMGWDGGDADDTLDGEVESARPLIEKGHQFNTLSGRETADLRNSWEMFERLLTQEQSYRSAGDRETW
NYDSNDVVELVVSVEDTGVGIPLHVQDRVFTPFMQADSSTSRTYGGTGIGLSISRCLVELMSGEMDFVSSPGV
GSTFTFTAEFQRVPANVPESILREGSNCLLQTPPLPTCFQGMRVLVVDGRQVRCEVTLRSPTPFLRLLSFSPQWLT
AVLAVLVIRVVYSPEFDFVHYSSSISADIEMIFVDKDAWGPGTGMNFRSLLSECYMESPSSYHNARASYQYRLTA
GRLPIGFQVHSMQKMVLVATSMNGLELEMVKASGFVDTVIRKPLRASLVAACLKQILGVGDGRDPGQELRGG
VNSLQNLLSGKRILVVDDNPVNRKVAAGALNKYGAQVECVHSGRAAIQKLKPPHNFDACFMDVQMPEMDGF
QATGLIRKAETLAADQWLAAGLPTFGVRFHVPILAMTADVIQATSEECMRCGMDGYVAKPFDEHQLYQAVAK
HFFQAN
>Potri.014G164700
MSCTGGGAGVFVKFSRLFVKRHRWELVKMSINCKLSGSNGTSQESFKLRKSKEVLHETNSARKWKRKFLLLWF
LGVAVTIGSIWLLFSFDSGALGRKGQSLDSCEEGAQVLLRHFNVSKNQLHALGSLFSDSDQVASLDCTKEPGPE
MLINDGIACALKVPCSKKQEFQQHIRWVAEDVGPNGKCPVQDENEFRKLDRSLLDESASFVSQSTISSISQDFG
KRREVDCAEDHCKLFSFDLVKECWWVLVGMIVSCILLGYNLKFWRKQNQKLVQLEPVPQQRQQLLQMNQHQ
LSHSPPRGAGKWRKKLLIIFVLLGVLVSIWLFWHLHEKIISRREETLANMCDERARMLQDQFNVSMNHVHALAI
LVSTFHHGKNPSAIDQKTFGEYTKRTDFERPLTSGVAYALKVPHLERKQFEEQHGWTIKKMGTEDQTLVQDCIP
DKLDPAPIQDEYAPVIFSQETVSHIVSIDMMSGKEDRENILRARASGKGVLTSPFKLLKSNHLGVVLTFAVYNTDL
PDATPEQRIDATVGYLGASYDVPSLVEKLLHQLSSKQTIVVNVYDTTNATAPILMYGTDVTDTGLLHVSSLDFGDP
LRKHEMHCRFKHKPPLPWPAINASVGLLVITLLVGHIFHAAINRIAKVEEDYREMMELKARAEAADVAKSQFLAT
VSHEIRTPMNGVLGMLQMLMETDLDVNQMDYANTAHTSGKDLIALINEVLDQAKIESGRLELEAVPFDLRSVL
DNVLSLFSGKSNEKGIEKLAVYVSDRLPEVVIGDPGRFRQIITNLVGNSIKFTRDKGHVFVSVHLADEVRSPLDAR
DAVLKQGLELVQDTSSKVYDSLSGFPVVNRWKSWEKFKKSSCIDSRDEPEMIRLLVTVEDTGVGIPEDAQGNIFT
PFMQADSSTSRKYGGTGIGLSISKCLVDLMGGEIGFVSECGIGSTFSFIVSFRKGESTSLDTKWQPYDPAVLEVRG
GRALVIDERSVRAEVTKYHLQRLGITADVAPSLKSACAYLSSGYCTSIPADLPIVLIDKDAWDKESGIAFHHLLKMP
KKNDGTDIQVDLSKIFLLATTISSDERLELKTSGLVDNVLVKPLRLSVLIACFQEAFGSGKKSEVNRKKPPALQNLLR
GKQILVVDDNLVNRRVAEGALKKHGAIVTCVESGKAALEKLKPPHSFDACFMDFQMPEMDGFEATRQIRSME
SQFNEKIASGKASMELPGNVAYWHTPILAMTADVIQATNEECLKCGMDGYVSKPFEDEKLYNAVTRFF
>Potri.001G057400
MSLLHVYGFGLKVRHLLWMLCCWIVSVISMNWFINGGVLETQASLLGDGGKMWLKCLEKVSGNSCKIHHHY
YQYIGSKRISKTWWRKLLVAWIVGWITVSVWIFWYMSSQAFEKRKETLTSMCDERARMLQDQFNVSMNHV
QAMSILISTFHHAKNPSAIDQRTFARYTERTAFERPLTSGVAYAVRVLHSEREQFEKQQGWTIKRMDSFEQNPV
HKDDNAPKALEPSPIQEEYAPVIFAQDTVAHVVSLDMLSGTEDRENVLRARASGKGVLTAPFRLLKTKRLGVILTF
AVYKTDLPSNATPNERIQATDGYLGGIFDIESLVEKLLQQLASKQTILVNVYDTTNQSCPISMYGSNVSDDGLEHV
SALNLEDPFRKHEMRCRFKQKPPWPWLAITTSIGILVIALLIGYIFHATMNRIAKVEDDCHKMMELTKQAEAAD
VAKSQFLATVSHEIRTPMNGVLGMLHMLMDTDLDANQQDYVRTAQDSGKALVSLINEVLDQAKIESGKIELEE
MQFDLRAIMDDVLALFSGKAHEKGIELAVYVSDGVPEMLIGDPGRFRQIITNLMGNSIKFTKKGHIFLTVHPVEE
VMDSIDVETESSSLNTLSGLPVADRRRSCAGFKIFSREGSSHTLSPSSSDLVNLIVSVEDTGEGIPLEAQPRVFTPF
MQVDPSISRKYGGTGIGLSISKCLVGLMNGEIGFASIPDTGSTFTFTAVFRNGCSNSNDSKQQKQRIKNQCNTTP
SEFQDMTALVVDPKPVRANVSRYQIQRLGIHVELVSDLNQGLSIISNENRIFKMIFVEQEVWEKDSSISAHFVNN
LQKIERGVSSKLFLLGNSLSSSRTNTATSGAYTLSVITKPLKASMLAASLQRAMGGNKGNPRNGEHPSLSLCNHL

VGRKILIVDDNKVNLIVAAAALKKYGAEVICADSGKMAIKLLKPPHQFDACFMDIQMPEMDGFEATRRIRDME
SNGHIPILAMTADVIQATYEECQRCGMDGYVSKPFEAEQLYQEVSRFLQPTSNVNL
>Potri.003G171000
MSLLHVFGFGLKVGHLLWMLCCWIVSVISMNWFINGGILETKAGLLGDGGKMWLKCLEKVSGNSCKIHHLYY
QYIGSKRIRKTWWRKLLVAWIVGWITVSVWIFWYMSSQAFEKRKETLASMCDERARMLQDQFNVSMNHVQ
AMSILISTFHHGKNPSAIDQRTFARYTERTAFERPLTSGVAYAVRVMHSEREQFENQQGWTIKRMDTFEQSPVQ
KDDNVAKALEPSPIQEEYAPVIFAQDTVAHVVSLDMLSGTEDRENVLRARASGKGVLTAPFRLLKTNRLGVILTFA
VYKTDLPSNAMPNERIQATDGSSDLLMTPIRYLGGIFDIESLVEKLLQQLASKQTILVNVYDITNQSHPISMYGSN
VSDDGLEHVSALNFGDPFRKHEMRCRFKQKPPWPWLAITTSIGILVIALLIGYIFHATMNRIAKVEDDYNEMME
LKKRAEAADVAKSQFLATVSHEIRTPMNGVLGMLHMLMDTELDATQQDYVRTAQDSGKALVSLINEVLDQAKI
ESGKIELEAMQFDLRAIMDEVLALFSGKAHEKGVELAVYVSDGVPEMLIGDPGRFRQIITNLMGNSIKFTKKGHI
FLTVHLVEEVMDSIDVETESSSRNTLSGLPVADRCRSWVGFKTFNPEGSSHTLSPSSSDLINLIVSVEDTGEGIPLE
AQPRVFTPFMQVDPSISRKYGGTGIGLSISKCLVGLMNGDIGFVSIPDIGSTFTFTAVFSNGCSNSNDSKLQKQRL
KTQTNTMSSKFQGMTALVVDPKPVRAKVSRYQIQRLGIHVELVLDLNQGLSSISNENKVVNMVFIEQEVWDKD
SSISALFVNKLQKIVSGVSTKLFLLGNSLSSRTNTATSGVYTPSVITKPLKASMLAASLQRAMGGNKGNPCNGEH
PSLSLRKLLVGRKMLIVDDNKVNLMVAAAALKKYGADVVCADSGQKAIKLLKPPHKFDACFMDIQMPEMDGF
EATRRIRDMESNWHIPILAMTADVIQATYEECQRCGMDGYVSKPFEAEQLYHEVSRFLQPTSSANL
>Potri.008G137900
MALKMQQSYHHSVAVKVNDQQMGTKRGYTFIQANRTWLPKVLLLWVMAMALFSLTIYNGMDADNKVRRK
EVLGSMCDQRARMLQDQFSVSVNHVHALAILVSTFHYYKNPSAIDQETFAEYTARTAFERPLLSGVAYARRVIDS
ERHEFERQHGWTIKTMEREPSPIRDEYAPVIFSQETVSYIESLDMMSGEEDRENILRARATGKAVLTSPFRLLGSH
HLGVVLTFPVYKSKLPPSPTVAQRIEATAGYLGGAFDIESLVENLLGQLAGNQAILVNVYDITNSSDLLIMYGHQN
LDGDLSLLHESKLDFGDPFRKHLMTCRYHEKAPTSWTALSTAFLFFVIGLLVGYILYGAAIHIVKVEDDFHEMQEL
KVRAEAADVAKSQFLATVSHEIRTPMNGVLGMLALLLDTDLSSTQRDYAQTAQVCGKALIALINEVLDRAKIEAG
KLELEAVPFDIRSILDDVLSLFSEKSRNKGIELAVFVSDKVPEIVVGDPGRFRQIITNLVGNSVKFTERGHIFVKVHLY
ENAKAITDTKVDTCLNGGSNESVLTSGSQKFKTLSGCEAADDQNSWDVFKHFSDEDFRFDASINVMTNNEASE
DVGLMVCVEDTGIGIPLKAQGRVFMPFVQADSSTSRQYGGTGIGLSISKCLVELMGGQINFISRPEVGSTFSFTA
VFGTCKKNTFNNMEKRNAEELPSGFRGLKALVVDGNPVRATVTRYHLKRLGILAEVVSSLKLAAIGCGKNGSLTS
GRGKIHPDIILVEKDSWISGEDGVSSVWQLDSKQNGHAFKLPKMILLATNITNSEFDIAKGAGFADTVILKPLRSS
MVAACLLQVLGMGKKRSQGKGMPNGSSFLQSLLCGKRILVVDDNRVNRRVAAGALKKFGADAECAESGKEAL
KLLQPPHTYDACFMDIQMPEMDGFEATRRIRQMESQANEQMNGESMVEEGTARKVQWHIPILAMTADVIH
ATHDECLKSGMDGYVSKPFEEENLYQAVARFFDTKSTLKS
>Potri.010G102900
MALNIQQSYHHSVAVKMNGQQMGTKRGYTFIQANRTWLPKILLLWVMAMALFSLTIYNGMDADNRVRRKE
VLSSMCDQRARMLQDQFNVSVNHVHALAILVSTFHYYKNPSAIDQETFAEYTARTAFERPLLSGVAYAQRVVNS
ERLEFERQHGWTIKTMEREPSPIRDEYAPVIFSQETVSYIESLDMMSGEEDRENILRARASGKAVLTGPFRLLGSH
HLGVVLTFPVYKSKLPPSPTVAQRIEATAGYLGGAFDVESLVENLLGQLAGNQAILVNVYDITNSSDLLIMYGHQN
QDGDMSLLHESKLDFGDPFRRHLMTCRYHEKAPTSWTALTTTFLFFVIGLLVGYILYEAAIHIVKVEDDFHEMQD
LKVQAEAADVAKSQFLATVSHEIRTPMNGILGMLALLLDTDLSSTQRDYAQTAQVCGKALIALINEVLDRAKIEA
GKLELEAVPFHIRSIVDDVLSLFSEKSRNKGIELAVFVSDKVPEIVVGDPGRFRQIITNLVGNSVKFTERGHTFVKV
HLYEHAKATTDTKADTCLIGGSNESVLISGSQKFKTLSGCEAADDQNSWDVFKHLSDEDFRFDASINVMTSNEA
SENITLMVCVEDTGIGIPLKAQSRVFMPFVQADSSTSRHYGGTGIGLSISKCLVELMGGQISFISRPEVGSTFSFTA
VFSTCKKNAFTKMEKRNAEDLPSGFRGLKALVVDGKPVRAAVTRYHLKRLGILAEVVSNLKVAAGSCGKNGSLT
SGSKIQPDMILVEKDTWISGEDGVSNVWKLDWKQNGHAFKFPKMILLATNITNSEFDKAKAAGFADTVIMKPL

RASMVAACLLQVLGMGKKRSQGKCMPNGSSFLQSLLCGKKILVVDDNRVNRRVAAGALKKFGADVECADSGK
EALKLLQLPHTFDACFMDIQMPEMDGFEATRRIRQMESQANEQMNGESMVEGGTARKGQWHIPILAMTAD
VIHATHDECLKCGMDGYVSKPFEEENLYQAVARFFDSKSTLKS
>AT3G21510_AHP1
MDLVQKQKSLQDYTKSLFLEGILDSQFLQLQQLQDESNPDFVSQVVTLFFQDSDRILNDLSLSLDQQVVD
FKKVDPHVHQLKGSSSSIGAQRVKNACVVFRSFCEQQNVEACHRCLQQVKQEYYLVKNRLETLFKLEQQI
VASGGMIPAVELGF
>AT3G29350_AHP2
MDALIAQLQRQFRDYTISLYQQGFLDDQFTELKKLQDDGSPDFVSEVLSLFFEDCVKLISNMARALDTTG
TVDFSQVGASVHQLKGSSSSVGAKRVKTLCVSFKECCEAKNYEGCVRCLQQVDIEYKALKTKLQDMFNLE
KQIIQAGGIVPQVDIN
>AT5G39340_AHP3
MEKLCDYFFIYFCPNLMICKNLFIGIAISNKLYIFFFSLLLPSRRVERAMDTLIAQLQRRFCDFTISLYH
QGFLDDQFTELKKLQDECSPDFVAEVVTLFFEDCEKLISNMARALDQTGNVDFKLVGSSVHQLKGSSSSV
GAKRVKGLCVTLKECCDSQNYEGCVRCLQQVDIEYKTLKAKLQDLFNLEQQIVQAGGRIPQVDI
>AT3G16360_AHP4
MQRQVALIKQSLFDQGYLDEQFMELEELQDDANPNFVEEVSALYFKDSARLINNIDQALERGSFDFNRLD
SYMHQFKGSSTSIGASKVKAECTTFREYCRAGNAEGCLRTFQQLKKEHSTLRKKLEHYFQLARQAGPKET
ARRPK
>AT1G03430_AHP5
MNTIVVAQLQRQFQDYIVSLYQQGFLDNQFSELRKLQDEGTPDFVAEVVSLFFDDCSKLINTMSISLERP
DNVDFKQVDSGVHQLKGSSSSVGARRVKNVCISFKECCDVQNREGCLRCLQQVDYEYKMLKTKLQDLFNV
SFKLCENLLVLLVYSFY
>Pp1s105_12V6.1
MAEPKVVGKVSDAAAPASAVQAVEQVKVREAEAEVKVKEEEEEGECSVDELLDQHQELLDGMLSEGYLDDQF
SQLQMLQDESSPDFVEEVVTLFFDDTEKLLENLTESLKTDPVDFKVVDGHVHQFKGSSSSIGAQRVKNVCVAFR
HCCDAEDKKGCVDHLAKVKEEFNDVRSKLRKMLELEKRIMAAGGVLPFVEC
>Pp1s294_68V6.1
MGLEFKYVEDSSDEDEKMTASEEKWQDLEALQARHEELVNSMYREELLNYRFSELQELQDAENPNFVEEIITGH
MEECASCIEELEKALKTDPVDYHFCSPVWSYGCQIGNSNASIGAHQVAIWCGKFRDCVSREDKQGCLDALEKV
KEAFDILRPKLLTMLEFEREIAATGGTVYYMNCNNHV
>Potri.008G197600
MEVGQMQRAWVEYTKSLFREVGFLDAQFQQLQLLQDESNPDFVAEVVSLFFEDSERLLTD
LTSALEQQNIDFKKVDAHVHQFKGSSSSIGALRVKNDCIAFRNFCEEQNIEGCLRCLQQL
KQDYYLVKSKLEALIRLEQQIVAACGTIPMEELSS
>Potri.010G027100
MEVVQMQRAWVEYTKSLFREFSYSFFGWMKGFLDAQFQQLQLLQDESNPDFVAEVVSLFF
EDSERLLSDLTFALEQQSVDFKKVDAHVHQLKGSSSSIGAQRVKNDCIAFRSFCEEQNIE
GCQRCLQQVKQDYCLVKSKLEALIRLEQQIVAAGGSIPMEELSC
>Potri.001G189900
MERNQLRRQVALTRQSLFDQGFLDEQFIQLEELQDDANPNFVEEVVSLHYRDSARLISNI
EKALEKNPLDFNKLDGYMHQFKGSSSSIGAKKVKAECTLFREYCKAGNGEGCMRTFQQIK
KEYATLKRKLETYFQLARQAGPADTACRPNCVKYGYGLFPSWTLVQAQFCSQVPALSRDR
KKPNAGLYPEESLYSVGMECWSYRSYTSNLLWIQWNISQSIIRN

>Potri.001G465000
MASTPLRQQLSTMRQSFFDEGLLDHGQVSYLETLENEDDPDFIENIFTLFLRDSSKYIAS
IEKALETAPDDFTFVLERMMYRLKGSSASIGATKINDETNKLRGFCHEGDLESAKASLQK
LKAEHANFKQKLAVYVGIMKQVKLSEC
>Potri.006G236300
MDRKQLQHQVASTRRSLFDQGYLDDQFIQLEHLQDEANPNFVEEVVRLFYSDSVRLIQNI
EQAMINKPNIDFGKLDDYMHQFKGSSSSIGAKKVMKECSKFREYCYAGNIEGCIKTFQLL
KQEHATLRRKLETYFQLVKQAGLAESA
>Potri.009G146300
MGGPNIWEEQRNFVKNLHEQGILDSRFDEILDLPRENPQFVIDLVTKFCSDAENSIAALI
RYHNEPDINYPKVIDRAHQIKGASSCIGGHRMALASRELRYACEDKDKDRCFAAFCKTKD
EYQILKEKFNIILQVMLRKKNY
>Potri.001G191900
MLGWGVDRLRADMSRLLAILFHQGVLDEQFLQLQQLQDESSPNFVSEVVTIYFHESEKLL
RNLRGLLLDREFSDYKKMGIHLNQFMGSSSSIGAKRVRNVCVAFRAASEQNNRAGCLRAL
ELLEHEYCYLKNKLHELFQMEQQRVLAAGVRYPLHQQH
>Potri.003G032400
MLGWGVDRLRADMNRLLAMLFHQGVLDEQFLQLQQLEDESSPNFVSEVVNIYFHESEKLL
RNLRGLLMDREFSDYKKMGTHLNQLIGSSSSIGAKRVRNVCVSFRAASEQNSRAGCLRAL
ELLEHEYCYLKNKLHELFQMDQLRVLAAGVTYPPQH
>Potri.006G098200
MDLLNQLQRQYTDFTTLLYHEGFVDDQFTQLQKLQDESSPDFVVEVVSLFFEDCEKLVNN
MAKALEQQDVDFKQVDSHVHQLKGSSSSIGAARIKNVCIAFKTFCEAQNREGCLRCLQQV
NHEHTQLKTNLQSLFTLERQIVANGGSIPVMQ
>Potri.014G136200
MDLLSQLRGQIADFSAFLYREGFVDDQFTQLQKLQDESSPGFVVEVVSLFFEDCEKLVNN
MAKALEQQIVDFKQVDSHVHQLKGSSSSIGAARIQNVCIAFKTYCEGQNRDGCVRCLQQV
NHECIQLKNNLQALFKLEQQIVAAGGSIPVMQ
>Potri.016G113500
MDVVSQLQRQLADFLAPLYREGFVDDQFTQLQKLQDESSPDFVMEVVSLFFEDCEKLVNN
MAKALEQQVVDFKQVDSHVHQLKGSSSSIGAARIKNVCIAFKTFCEAQNREGCLRCLQQV
NHEYTQLKANLQTLFTLERQIVAAGGSVPAMQ
>Potri.013G028300
MDSVVQLQRQLVDYSAQLFNEGFLDDQFNQLQQLQDESNPDFVVEVVTLFFEDSERLLDE
LAKALEQQSVDYRKIDAHVHQLKGSSSSIGAQRVQKVCIGFRNFCEERNIEGCQKCLQQV
RHEYSLVKTKLETLFKLEQKILAAGGSIPWPM
>Potri.005G040400
MDSVVQLQRQLVDYTGQLFNEGFLDDQFNQLQQLQDESNPDFVVELVTLFFEDSEKLINE
LAKDLEQQSIDYRKIDAHVHQLKGSSSSIGVQRVQRVCIAFRNYCEERNIEGCQKCLQQV
KNEYSLVKTKLETLFKLEQQVLAAGGSIPWPM
>AT1G59940_ARR3
MNFEFIFLYLFRLVRYFLLPPSQKRKKGTYRFVSLIVTWNKKIFCKRNEVENDPPIKRFDIEEYNNENIK
QSREELVAISLFYLFISYSFLTMAKDGGVSCLRRSEMIGIGIGELESPPLDSDQVHVLAVDDSLVDRIVI
ERLLRITSCKVTAVDSGWRALEFLGLDDDKAAVEFDRLKVDLIITDYCMPGMTGYELLKKIKESTSFKEV

PVVIMSSENVMTRIDRCLEEGAEDFLLKPVKLADVKRLRSYLTRDVKVAAEGNKRKLTTPPPPPPLSATS
SMESSDSTVESPLSMVDDEDSLTMSPESATSLVDSPMRSPGLA
>AT1G10470_ARR4
MARDGGVSCLRRSEMMSVGGIGGIESAPLDLDEVHVLAVDDSLVDRIVIERLLRITSCKVTAVDSGWRAL
EFLGLDNEKASAEFDRLKVDLIITDYCMPGMTGYELLKKIKESSNFREVPVVIMSSENVLTRIDRCLEEG
AQDFLLKPVKLADVKRLRSHLTKDVKLSNGNKRKLPEDSSSVNSSLPPPSPPLTISPESSPPLTVSTESS
DSSPPLSPVEIFSTSPLSSPIDDEDDDVLTSSSEESPIRRQKMRSPGLD
>AT3G48100_ARR5
MAEVLRPEMLDISNDTSSLASPKLLHVLAVDDSMVDRKFIERLLRVSSCKVTVVDSATRALQYLGLDGEN
NSSVGFEDLKINLIMTDYSMPGMTGYELLKKIKESSAFREIPVVIMSSENILPRIDRCLEEGAEDFLLKP
VKLADVKRLRDSLMKAEERAFKNIMHKRELEANDIYSQLKRAKI
>AT5G62920_ARR6
MAEVMLPRKMEILNHSSKFGSPDPLHVLAVDDSHVDRKFIERLLRVSSCKVTVVDSATRALQYLGLDVEE
KSVGFEDLKVNLIMTDYSMPGMTGYELLKKIKESSAFREVPVVIMSSENILPRIDRCLEEGAEDFLLKPV
KLSDVKRLRDSLMKVEDLSFTKSIQKRELETENVYPVHSQLKRAKI
>AT1G19050_ARR7
MAVGEVMRMEIPAGGDLTVTTPELHVLAVDDSIVDRKVIERLLRISSCKVTTVESGTRALQYLGLDGGKG
ASNLKDLKVNLIVTDYSMPGLSGYDLLKKIKESSAFREVPVVIMSSENILPRIQECLKEGAEEFLLKPVK
LADVKRIKQLIMRNEAEECKILSHSNKRKLQEDSDTSSSSHDDTSIKDSSCSKRMKSESENLFSLL
>AT2G41310_ARR8
MVMETESKFHVLAVDDSLFDRKMIERLLQKSSCQVTTVDSGSKALEFLGLRVDDNDPNALSTSPQIHQEV
EINLIITDYCMPGMTGYDLLKKVKESAAFRSIPVVIMSSENVPARISRCLEEGAEEFFLKPVKLADLTKL
KPHMMKTKLKKESEKPVAIEEIVVSKPEIEEEEEESSVIEILPLHQEIESEQLEPMLSSNKRKAMEEVVS
TDRSRPKYNDITTSV
>AT3G57040_ARR9
MGMAAESQFHVLAVDDSLFDRKLIERLLQKSSCQVTTVDSGSKALEFLGLRQSTDSNDPNAFSKAPVNHQ
VVEVNLIITDYCMPGMTGYDLLKKVKESSAFRDIPVVIMSSENVPARISRCLEEGAEEFFLKPVRLADLN
KLKPHMMKTKLKNQKLEEIETTSKVENGVPTAVADPEIKDSTNIEIEILPLQQDLLLVQQEEQTLSINNK
RKSVEEGISTDRARPRFDGIATAV
>AT1G74890_ARR15
MALRDLSSSLSSSSSPELHVLAVDDSFVDRKVIERLLKISACKVTTVESGTRALQYLGLDGDNGSSGLKD
LKVNLIVTDYSMPGLTGYELLKKIKESSALREIPVVIMSSENIQPRIEQCMIEGAEEFLLKPVKLADVKR
LKELIMRGGEAEEGKTKKLSPKRILQNDIDSSPSSSTSSSSSSHDVSSLDDDTPSSKRIKLESRG
>AT2G40670_ARR16
MNSSGGSCSSLMDVVAYDHHLHHGHDEELHVLAVDDNLIDRKLVERLLKISCCKVTTAENALRALEYLGL
GDQNQHIDALTCNVMKVSLIITDYCMPGMTGFELLKKVKESSNLREVPVVIMSSENIPTRINKCLASGAQ
MFMQKPLKLADVEKLKCHLMNCRS
>AT3G56380_ARR17
MNKGCGSGSDSCLSSMEEELHVLAVDDNLIDRKLVERILKISSCKVTTAENGLRALEYLGLGDPQQTDSL
TNVMKVNLIITDYCMPGMTGFELLKKVKQESSNLKEVPVVILSSENIPTRINKCLASGAQMFMQKPLKLS
DVEKLKCHLLNCRS
>AT3G16857_ARR1
MMNPSHGRGLGSAGGSSSGRNQGGGGETVVEMFPSGLRVLVVDDDPTCLMILERMLRTCLYEVTKCNRAE
MALSLLRKNKHGFDIVISDVHMPDMDGFKLLEHVGLEMDLPVIMMSADDSKSVVLKGVTHGAVDYLIKPV

RMEALKNIWQHVVRKRRSEWSVPEHSGSIEETGERQQQQHRGGGGGAAVSGGEDAVDDNSSSVNEGNNW
R
SSSRKRKDEEGEEQGDDKDEDASNLKKPRVVWSVELHQQFVAAVNQLGVEKAVPKKILELMNVPGLTREN
VASHLQKYRIYLRRLGGVSQHQGNLNNSFMTGQDASFGPLSTLNGFDLQALAVTGQLPAQSLAQLQAAGL
GRPAMVSKSGLPVSSIVDERSIFSFDNTKTRFGEGLGHHGQQPQQQPQMNLLHGVPTGLQQQLPMGNRMS
IQQQIAAVRAGNSVQNNGMLMPLAGQQSLPRGPPPMLTSSQSSIRQPMLSNRISERSGFSGRNNIPESSR
VLPTSYTNLTTQHSSSSMPYNNFQPELPVNSFPLASAPGISVPVRKATSYQEEVNSSEAGFTTPSYDMFT
TRQNDWDLRNIGIAFDSHQDSESAAFSASEAYSSSSMSRHNTTVAATEHGRNHQQPPSGMVQHHQVYADG
NGGSVRVKSERVATDTATMAFHEQYSNQEDLMSALLKQV
>AT4G16110_ARR2
MVNPGHGRGPDSGTAAGGSNSDPFPANLRVLVVDDDPTCLMILERMLMTCLYRVTKCNRAESALSLLRKN
KNGFDIVISDVHMPDMDGFKLLEHVGLEMDLPVIMMSADDSKSVVLKGVTHGAVDYLIKPVRIEALKNIW
QHVVRKKRNEWNVSEHSGGSIEDTGGDRDRQQQHREDADNNSSSVNEGNGRSSRKRKEEEVDDQGDDKE
DSSSLKKPRVVWSVELHQQFVAAVNQLGVDKAVPKKILEMMNVPGXTRENVASHLQKYRIYLRRLGGVSQH
QGNMNHSFMTGQDQSFGPLSSLNGFDLQSLAVTGQLPPQSLAQLQAAGLGRPTLAKPGMSVSPLVDQRSI
FNFENPKIRFGDGHGQTMNNGNLLHGVPTGSHMRLRPGQNVQSSGMMLPVADQLPRGGPSMLPSLGQQP
ILSSSVSRRSDLTGALAVRNSIPETNSRVLPTTHSVFNNFPADLPRSSFPLASAPGISVPVSVSYQEEVNSSDAKGG
SSAATAGFGNPSYDIFNDFPQHQQHNKNISNKLNDWDLRNMGLVFSSNQDAATATATAAFSTSEAYSSSSTQR
KRRETDATVVGEHGQNLQSPSRNLYHLNHVFMDGGSVRVKSERVAETVTCPPANTLFHEQYNQEDLMSAFLK
QEGIPSVDNEFEFDGYSIDNIQV
>AT4G31920_ARR10
MTMEQEIEVLDQFPVGMRVLAVDDDQTCLRILQTLLQRCQYHVTTTNQAQTALELLRENKNKFDLVISDV
DMPDMDGFKLLELVGLEMDLPVIMLSAHSDPKYVMKGVKHGACDYLLKPVRIEELKNIWQHVVRKSKLKK
NKSNVSNGSGNCDKANRKRKEQYEEEEEEERGNDNDDPTAQKKPRVLWTHELHNKFLAAVDHLGVERAVP
KKILDLMNVDKLTRENVASHLQKFRVALKKVSDDAIQQANRAAIDSHFMQMNSQKGLGGFYHHHRGIPVG
SGQFHGGTTMMRHYSSNRNLGRLNSLGAGMFQPVSSSFPRNHNDGGNILQGLPLEELQINNNINRAFPSF
TSQQNSPMVAPSNLLLLEGNPQSSSLPSNPGFSPHFEISKRLEHWSNAALSTNIPQSDVHSKPDTLEWNA
FCDSASPLVNPNLDTNPASLCRNTGFGSTNAAQTDFFYPLQMNQQPANNSGPVTEAQLFRSSNPNEGLLM
GQQKLQSGLMASDAGSLDDIVNSLMTQEQSQSDFSEGDWDLDGLAHSEHAYEKLHFPFSLSA
>AT1G67710_ARR11
MEKSGFSPVGLRVLVVDDDPTWLKILEKMLKKCSYEVTTCGLAREALRLLRERKDGYDIVISDVNMPDMD
GFKLLEHVGLELDLPVIMMSVDGETSRVMKGVQHGACDYLLKPIRMKELKIIWQHVLRKKLQEVRDIEGC
GYEGGADWITRYDEAHFLGGGEDVSFGKKRKDFDFEKKLLQDESDPSSSSSKKARVVWSFELHHKFVNAV
NQIGCDHKAGPKKILDLMNVPWLTRENVASHLQKYRLYLSRLEKGKELKCYSGGVKNADSSPKDVEVNSG
YQSPGRSSYVFSGGNSLIQKATEIDPKPLASASLSDLNTDVIMPPKTKKTRIGFDPPISSSAFDSLLPWN
DVPEVLESKPVLYENSFLQQQPLPSQSSYVANSAPSLMEEEMKPPYETPAGGSSVNADEFLMPQDKIPTV
TLQDLDPSAMKLQEFNTEAILRSLNWELPESHHSVSLDTDLDLTWLQGERFLANTGLQFQDYSSSPSLLS
ELPAHLNWYGNERLPDPDEYSFMVDQGLFIS
>AT2G25180_ARR12
MTVEQNLEALDQFPVGMRVLAVDDDQTCLKILESLLRHCQYHVTTTNQAQKALELLRENKNKFDLVISDV
DMPDMDGFKLLELVGLEMDLPVIMLSAHSDPKYVMKGVTHGACDYLLKPVRIEELKNIWQHVVRSRFDKN
RGSNNNGDKRDGSGNEGVGNSDQNNGKGNRKRKDQYNEDEDEDRDDNDDSCAQKKQRVVWTVELHKKF
VAAVNQLGYEKAMPKKILDLMNVEKLTRENVASHLQKFRLYLKRISGVANQQAIMANSELHFMQMNGLDGF
HHRPIPVGSGQYHGGAPAMRSFPPNGILGRLNTPSGIGVRSLSSPPAGMFLQNQTDIGKFHHVSSLPLNHS

DGGNILQGLPMPLEFDQLQTNNNKSRNMNSNKSIAGTSMAFPSFSTQQNSLISAPNNNVVVLEGHPQATPP
GFPGHQINKRLEHWSNAVSSSTHPPPPAHNSNSINHQFDVSPLPHSRPDPLEWNNVSSSYSIPFCDSANTLSSP
ALDTTNPRAFCRNTDFDSNTNVQPGVFYGPSTDAMALLSSSNPKEGFVVGQQKLQSGGFMVADAGSLDDIV
NSTMKQEQSQGDLSGGDLGYGGFSSLRTCI
>AT2G27070_ARR13
MAFAQSVYNQSSVLKINVMVVDDNRVFLDIWSRMLEKSKYREITVIAVDYPKKALSTLKNQRDNIDLIIT
DYYMPGMNGLQLKKQITQEFGNLSVLVMSSDPNKEEESLSCGAMGFIPKPIAPTDLPKIYQFALTYKRNG
KSTLSTEQNQKDANVSVPQQIMLVPEQAYVLKTKKKNCSSKSDTRTVNSTNVSHVSTNGSRKNRKRKPKG
GPSDDGESLSQPPKKKKIWWTNPLQDLFLQAIQHIGYDKVVPKKILAIMNVPYLTRENVASHLQKYRLFV
KRVVHQGRFSMLSDRGKDSMFRQTHIKEPYVNYYTPSTSWYETSLNNRSFYSESVHGHSRLLSEAREPVR
YNQMSYNYMNRNISFENQPSQNEETRTVFEPPVMANKISQTSQVLGFGQLGPSAISGHNFNTNMMSSYGS
LTPNQPGTSHFSYGMQSVLNNENATYNPQPPANATTQPNLDELPQLENLNLYNDLGNTSELPYNISNFQS
DDNKKQGEEDGDWTFVNINQDQSNGESSNTIATPETNTPNFNINPNQNQGQAVPEFTDWSFLDQQELVDD
DFMNSLFNNDMN
>AT2G01760_ARR14
MPINDQFPSGLRILVVDDDTSCLFILEKMLLRLMYQGLILYQSSHLLIDFLKKVLTFLFYLLFVVTICSQ
ADVALTILRERKDSFDLVLSDVHMPGMNGYNLLQQVGLLEMDLPVIMMSVDGRTTTVMTGINHGACDYLI
KPIRPEELKNIWQHVVRRKCVMKKELRSSQALEDNKNSGSLETVVVSVSECSEESLMKCRNKKKKKKRSV
DRDDNEDDLLLDPGNSKKSRVVWSIELHQQFVNAVNKLGIDKAVPKRILELMNVPGLSRENVASHLQKFR
LYLKRLSGEASQSNDSESTKRYENIQALVSSGQLHPQTLAALFGQPIDNHHSASFGVWIPNDNLGRSQNE
HFSVDVSSASNRPVSVAVHGLSSSANFRQRGDVNNNRIRQGYGSNVNEESWILERSSRQR
>AT5G58080_ARR18
MEFGSTEDGRHDKFPVGMRVLAVDDNPTCLRKLEELLLRCKYHVTKTMESRKALEMLRENSNMFDLVISD
VEMPDTDGFKLLEIGLEMDLPVIMLSAHSDYDSVMKGIIHGACDYLVKPVGLKELQNIWHHVVKKNIKSY
AKLLPPSESDSVPSASRKRKDKVNDSGDEDDSDREEDDGEGSEQDGDGSGTRKKPRVVWSQELHQKFVSA
VQQLGLDKAVPKKILDLMSIEGLTRENVASHLQKYRLYLKKIDEGQQQNMTPDAFGTRDSSYFQMAQLDG
LRDFTAARQIPSSGLLSRSHLTKLQPPMYSSINLQGMNSSSFIQQGHHQNSSNSANPFGTYHSTLSPRIQ
NVNLFQRTSSPLEPLQFPRSKSYIGDFKGLGDRAIGGSFLDTCMPFGSSSTSLPSASTNPLMLQANYTQP
LHIASDGIQPCIEGTPSNSASPNISFQGLSRFPGHSWQGNLNTTRFPPSSLPLNLAFLPDQVTCAGNNLG
DCTSLVSAENPGGEMQCDPQLLGGFMQNVNPLGGQKWEQQNCTMLNNPFGNIEYPLPADNMVFRDNNST
RSKGLDESLMNPIDNSQEYVGKATTMLDPEMKSGKPENDNQHDVFDDIMNEMMKQEENNGMVPVATRFG
FDSFPPP
>AT1G49190_ARR19
MLVGKISGYEDNTRSLERETSEITSLLSQFPGNTNVLVVDTNFTTLLNMKQIMKQYAYQVSIETDAEKAL
AFLTSCKHEINIVIWDFHMPGIDGLQALKSITSKLDLPVVIMSDDNQTESVMKATFYGACDYVVKPVKEE
VMANIWQHIVRKRLIFKPDVAPPVQSDPARSDRLDQVKADFKIVEDEPIINETPLITWTEEIQPVQSDLV
QANKFDQVNGYSPIMNQDNMFNKAPPKPRMTWTEVIQPVQSNLVQTKEFGQLNDYSQIMNQDSMYNKAA
TKPQLTWTEEIQPVQSGLVQANEFSKVNGYSQSMNQDSMFNKSATNPRLTWNELLQPVQSDLVQSNEFSQF
SDYSQIMNEDNMFNKAAKKPRMTWSEVFQPVQSHLVPTDGLDRDHFDSITINGGNGIQNMEKKQGKKPRK
PRMTWTEELHQKFLEAIEIIGGIEKANPKVLVECLQEMRIEGITRSNVASHLQVKKKTHTLNIKHRINLE
ENQIPQQTQGNGWATAYGTLAPSLQGSDNVNTTIPSYLMNGPATLNQIQQNQYQNGFLTMNNNQIITNPP
PPLPYLDHHHQQQHQSSPQFNYLMNNEELLQASGLSATDLELTYPSLPYDPQEYLINGYNYN
>AT3G62670_ARR20
MSVFSNILDENSRNLRNEIPCDDGIASPINDDDEEFLTKSNRVLLVGADSNSSLKNLMTQYSYQVTKYES

GEEAMAFLMKNKHEIDLVIWDFHMPDINGLDALNIIGKQMDLPVVIMSHEYKKETVMESIKYGACDFLVK
PVSKEVIAVLWRHVYRKRMSKSGLDKPGESGTVESDPDEYDDLEQDNLYESNEEGSKNTCDHKEEKSPTK
KPRMQWTPELHHHKFEVAVEKMGSLEKAFPKTILKYMQEELNVQGLTRNNVASHLQKYRQSSKKTCTPQEP
QEDFVWGNAGPDVTLAASKTLLSSHATPSYLINNQAAPRGSYFMNNIPYPSTSCLPVNNNNCFMTNPSTY
IDQFQHQLQQQQQHQQYQSTLNSISAMLTKQESRHVPSSAMENSEPLMIYNSNLPFGIDECFPPAGFNIF
DQIGHN
>AT5G07210_ARR21
MASAQSFYNQSSVLKINVMVVDDDHVFLDIMSRMLQHSKYRDPSVMEIAVIAVDDPKKALSTLKIQRDNI
DLIITDYYMPGMNGLQLKKQITQEFGNLPVLVMSSDTNKEEESLSCGAMGFIPKPIHPTDLTKIYQFALS
NKRNGKSTLSTEQNHKDADVSVPQQITLVPEQADVLKTKRKNCSFKSDSRTVNSTNGSCVSTDGSRKNRK
RKPNGGPSDDGESMSQPAKKKKIQWTDSLHDLFLQAIRHIGLDKAVPKKILAFMSVPYLTRENVASHLQK
YRIFLRRVAEQGLYSMLSDRGIDSMFRQTHIKEPYFNYYTPSTSWYDTRLNNRSFYSKPVHGFGQSKLLS
TTREPVCFNQMPYNYMNRSSTYEPHRIGSGSNLTLPIQSNLSFPNQPSQNEERRSFFEPPVMANKIAQTS
QVLGFGQLGPSAISGHNFNNNMTSRYGSLIPSQPGPSHFSYGMQSFLNNENVTYNPQPPANATTQPNLDE
LPQLENLNLYNDFGNTSELPYNISNFQFDDNKHQQGEADPTKFELPAAKFSTELNHEDDGDWTFVNINQG
QSNGETSNTIASPETNTPILNINHNQNQGQDVPEFNDWSFLDPQELVDDDFMNSLFNNDMN
>Pp1s10_348V6.1
MEALVQSVSNFEGKMESGMLSSYHDLPWKVLLPKERAKVQSKWKAKSRKVRDQGKQMSAGVGSAKTCESST
HTVGTGNQKVEPNSHDLPPVENSDSDQSRAEDPRSEETSAESVLSMSNPEYHVLAVDDSIIDRKVIERLLKTSSY
KVTTVNSALRALEYLGLSESCSTSVKANKVTVNLIMTDYCMPEMTGYDLLKIVKAETSAFKELPVIVMSSENDSN
RIKRCLEEGAKEFLIKPVQIEDVQRLHGHIRLASASSGSSDSSNTSTCAKRKAPDGLQPNSPVRCRTGIRISFNFQG
WTRDDWRNYRWTEALLVEDFSYKFCALVNILVSNLKMKYRIDRRRFQDLDVMKCRL
>Pp1s10_378V6.1
MVRKTLEAEVRGECGISHPPKDLKPPKPAEADVLGIPGLLHQTSGGSKPKKAFKLVGQTKLPDMGLDVLKKTRK
RGNSDGATTIPSPAQFFEDTLQKKVVGETRAETISLLSASLDNFCPDLVQKNLLLSELGAIKPAPVNTFQYHVLAV
DDSSIDQRVIERLLKTSSYRVTTVNSAVRALDVLGLSESRSASSTRLDINLIMTDYCMPEMTGYDLLKRVKAQTSA
LKEIPVVLMSSENDSNRIERCLAEGAEEFIIKPVKMADVKRLHGLLKRPSTRSSSETGESSGFTCGKRKADALDLH
EVSSPQRLLQFNEYFVS
>Pp1s12_62V6.1
MVRKSLEPEVSNESIVLDSIQDSKPPKVAETKLQDMGFDKPKKARQSYSETSKATAPPTCTEDPVQRKVVGETTA
ESISLISASIESYCAESVQKELRSDLVGIKPVPVVSSFQYHVLAVDDSRIDQRVIEKLLKTSSYKVTTVNSALRALELL
GLSDSSPSSIKKLDINLIMTDYCMPEMTGYDLLKRVKADTSALKEIPVVVMSSENDSNRIERCLAEGAEEFIIKPV
QMADVKRLRGHIRPVHTTSDTSSGTASENSGSTCGSKRKMVEPTDTSEVNSPERRPRLSEESVA
>Pp1s62_37V6.1
MAVDSSMNSEAACSNSGRFFSVNSRSIEIMAQCVSNLEGKAECSVVSSYHDSKSWEFLKPAERTKFKSISRDKS
RKLRDCGKPQGGGVGPSNTSQSTVRCVGTTDVRAEGYGVYALPVENSNSEESRAGDPQFEVTSAESALSKSNT
EYHILAVDDSRIDQKVIERLLKSASYKGTELTTVNSALRALKYLGLSESCASPVMPDRNAVHLIMTDYCMPEMTG
YELLKIVKAETSAFKDVPVILMSSENDSSRIERCLAEGAEEFIIKPVQMKDVQRLQGHIRSSGPSSGFSVTPNSLVC
RKRKSPDGLKPNSPVKRPRFDGVAVA
>Pp1s94_88V6.1
MDAMVQSVSKFEGTMEPSMLSPYHDMKPWKVLLPAERAKLQSKCREKSRNMCGVGSARTGERVGTGDQK
AEANGHVLQKFDSESSGSEDPRSEETSSDSVPNFSNSEYHVLAVDDSIIDRKVIERLLKTSSYKVTTVNSALRALEY
LGLSESCSSSGKPNKGAVNLIMTDYCMPGMTGYDLLKIVKAETSAFKELPVIVMSSENDSNRIKSCLAEGAEDFLI
KPVQLEDVQRLHGHMRSSAASSGSNESSNSSVCLKRKSSDGLQPNSPVRRPRYSGLAVA

>Pp1s172_31V6.1
MSVHPSALAAMVRKSLKPEVPAGSVKLNSFLVSKPRKVSEFAKHKKLKETGSKKSKKTRQSNSEPSIAKPQLSSSA
EDPVQRKLVGEKTAESINLISASIESYCADPVHNDLLSELVGTKPVPMISSSQYHVLTVDDSRIDQRVVEKLLMTSS
FKVTTVNSALRALELLGLSDSCPSSVKKPDINLIMTDYCMPEMTGYDLLKRVKADTSALKEIPVVVMSSENDSIRI
ERCLAEGAEEFIIKPVQMADVKRLRGHLRPVNSTSGSSSATASGSSDFTCGSKRKAESSDPQEVNSYERKPHPSE
VSVA
>Pp1s400_38V6.1
MTTSSTAQNIEETLQTKVRGETEAETMSLLSASIENRHPDSVKGKILRTELGAVKPESVRTKFQYHVLAVDDSMV
DQRVIEKLLKPLHAVTTVDSAVRALDVLGLSETRPSSSKVLHFNSFVQIKKLDINLIMTDYFMPEMTGYDLLKRVK
AKSSALKEIPVVLMSSENDSHRIRRCLAEGADAFLTKPVQLADVKRLRGYIRRQSSPSSSSGGTGGKINGDAVDH
RREVS
>Pp1s31_291V6.1
MVVMKPVNVRGVADDRMERLAHPEGFSPAGLKVLVVDDEPLALMIVERMLQICQYTVTTC
NSATKALAMLRENRNYFDLVISDVYMPDMDGFKLLEAIGLELDLPVIMMSGDGETDSVMK
GIRHGACDYLLKPVRLKELINIWQHVVRKLVTPRDIPKEESGEWDEFPKHQDNADFDSTA
RKRKERSEDVAQLVEDVNNLKKARVVWSAELHQQFVNAVNYLGVDKAVPRKILDIMNVQG
LTRENVASHLQKYRLYLKRLIGVTPQPYPVASFQAAENGTFGGTMQIQPGGRPAASTSTK
GLNLGGGASNSSIGGLSRSGVQIDAATLNALVQLQSLQQQKQLGPTAARSGLTTDVVNSQ
IPADPTGLQRLDSFELDLLMKTQHDASRQRGSGGSTDLADLLNCSKDALPYMSNLRGQCE
LKPAVMGSTNPLVGSEPMTAMSMSNLRGVDDGGPSLGSTEAGRSHMVNKLGRDFSNSSPA
FFSSTSRSNGGAVSDSSFSTNYGDIPDGPPSFLVASPPDLSLLGQLQAFDQVEFDYGQLS
QPRRSMSYSSKT
>Pp1s10_278V6.1
MGGGHLSSRSGDDMAEGNLGPTMTSLQQQPLPLIPNPHNSRPCYSATNSSSSNARGANLA
KLDTPDAFPAGLRVLVVDDEPVTLMVLEKMLTNCSYRVTTCGRATDALCLLREDIDKFDL
VISDVNMPDMDGFKLLELVGLEMDLPVIMMSGNGETSAVMKGITHGACDYLLKPVRQKEL
RNIWQHVVRKRRQDVKIARETKSVEEGGVCVREKRTGPDDVDYTSSATGDTGDGNWRKKR
KGENQTFKDETEEDVEQENDDPSTMKKPRVVWSVELHQQFVSAVNQLGIDRAVPKRILEL
MGVHGLTRENVASHLQKYRLYLKRISGVSNQQSNMSAHFGGPEPFSMLSPDMSVSIANGQ
LNPQALAQFHMLGRMNSSNGVGFSGGLDPMLSHQIFLQDLPRPPHLNNMLKRNTGMLSPL
PNAMPHLEPQLAECHHSSHHLPVMNELEDFSTNSKVFPQMNGNLDVSIASLGVGNGGLGS
NPSNDTLLMHMLQSRTAQQGSALSPTLPQPRGGLTPTHLLSNDVGFASVGSLPNLAPAVG
LSAITGSGGARDLSPSAVGTGGSLSSPLGPLVRRPLMVEEPSNLVNFSMAPSVQSPKPGG
GAGVNESLDQQPTMWGLYNPLTQLNPGHSRGLSQDNFPWSGLAVNLGLGDSQSLSVGMSS
GLGAQFSPQGQDHGIGFASPGQGSYNRQNVSFPVSSALDGRMVRSSYEP
>Pp1s16_393V6.1
MGGGYLSSTVNMGESRDGGSPAMATLQQQQKHQPLNPNHQNPRNRSNSSPTNCYSNTAWG
AKPAKLDTPDEFPVGMRVLVVDDNPTCLMILEQMLVRCAYRVTTCGKATEALSMLREDIG
KFDVVISDVDMPDMDGFKLLELVGLEMDLPVIMVSGNGETSAVMKGITHGACDYLLKPVR
IEELRNIWQHVVRKKRREVKAVATKSVEEAGGCERPKRGGGADDADYTSSATDTTDSNWK
LTKRRKGEFKDENEEDNEQENDDPSTLKRPRVVWSVELHQQFVSAVNQLGIDKAVPKRIL
ELMGVQGLTRENVASHLQKYRLYLKRLSGVTSQQGNMSAHFGGSDPFCMMPPDMSLANGQ
LTPQALAKFHMLGRMNATNGIGFSGGGLDPGMNQMFLQDLPRPPQLNSMLRNNTGLLASV
PNGLQHLEQLSEPHHVHVVNELEHYPSNTKVYPQLNGNLDVSVGPLGAANGNLASNPNSD

TLLMHILHSRASQQGVGSPSTLPQPRCGLNPTHLLSNDINFAPVGSLPNLAGSLGPAVGL
SAIPGSAGGRDLSPSVGGSGASLSSPLGSLVRRPLMAEEQSNPVNSTNGTYSMAHSGQSP
KPSGDTLPTPLNEGLEQQQPLWALYQNPMNQLSHGPSQGFPHDSLQWSVLTENLSFGDMG
QSLSAGLISQFSSQGQDNGIGFAPPSQRGSYTRQSVSFPASSALDGRMVRSSYEP
>Pp1s42_161V6.1
MERLAHPEGFSPAGLKVLVVDDDNVILKILERMLRECKYAVTTCNSATKALAMLRENRSY
FDLVISDVYMPDMDGFKLLEAIGLELDLPVIMMSRDGEIDSVMRGIKHGACDYLLKPVRL
EELRNIWQHVVRKLVSPILVSKEESGELDEYPKHQDNADFDATSRKRKERLEDETQLVED
VNNLKKARIVWSPELHQQFVNAVNYLGVDKAVPRKILDIMNVQGLTRENVASHLQKYRSY
LKRLIGVTPQPHPVASFQAAENGTFGGTMQIQPGGRPTASSSTKGLNLGGAAAISSISGL
NRGGSQIDAATLNTLVQLQSLQQKQLGTTASRVGLTTDLVSLQTPADPTGPQMLDSFELD
LLMKAQHDSRQRGGGGTDYTALLSGSKDTLPNMSSLRGHVELKPAAMGSTNLLAGSEPMT
AMSMSNLRGSDDLGPGLGGVDVGRSHLVNKWGGEFSNSSAFSSLTSRSNGGAVSESSFSI
DYGDIPDVPPEFLVASPSDLSLLGQLQAFDQGELDYDQLSQPRWSMSYSSKA
>Pp1s362_27V6.1
MGAEGGMQRAIVLSWSIATSGGNVSCRVGMGESRDGGSQVMVAPQQLQLPLKRSHQNQNN
RQCSGAINGASNSVRGANPAMLDTPDEFPVGMRVLVVDDDPTCLVLLERMLILCSYRVTT
CGRATDALLLLREDIGKFDLVISDVDMPDMDGFKLLELVGLEMDLPVIMMSGNGETSAVM
EGVIHGACDYLIKPVGIKELRNVWQHVVRKKRQEVKAVSMKSIEEAGGCERQKRAGGADD
ADYTSSATDTTDGNWKLSKRRKGEFKEENEDDNEENDDPSTMKKPRVVWSVELHQQFVSA
VNQLGIDKAVPKRILELMGVQGLTRENVASHLQKYRLYLKRLSGVTNQQCSMSAHFCGTE
PSSMISPDMGIANGQLTPQALAQLHMLGRMNSSNVIGFSGGLDPTLSHQFFLQDLPRPPH
LKDMLKNNAGFLSSFPNGLQHLDQLSEPHHLSVVNDLEDYPTNTKVFPQLNGNPGMSIGS
VGIAETNFGSNSNGDSLLMHILHSRAAHQGGGSSSNLPQPRAGLSPSHPLSNDINFAHVG
SLPNLAGNLVPTAELSSIPGSAGGRDLSSSVGFTGASLSSPLGSIVRGPLAAEEPSNLVN
STSGTFSCMVPSVQSSILRGVALHTSLNEGLDQQLPMWALYQNPVNRLSHGNSQGLSHDT
LQWSGLTENLGLGDMGQSLSAGLSSQYSSQSQDHGIGFAARSQRIAYTQQNVGFPVSSAL
DSRMVRSSYEPEGFQGVL
>Potri.010G037800
MSSNSIASNRWMSEKMDGFDPSPNSNSDNEEEGVHVLAVDDSLVDRKVIERLLKISSCKVTAVDSGWRALKLL
GLLDEEDKSSSSSSSSSAGFDVLKVDLIITDYCMPGMTGYELLKKIKQESTTFREIPVVIMSSENVVARIDRCLEEG
AEDFIVKPVKLSDVKRIRDYMASREVRSVQNQEERSSSNINKRKLQECFDLSLSSSPPSISSSSSSSLSYPSRSPSLTP
SPPSLFSSSAPCSPSSLDSPTRRLKMTGFD
>Potri.008G193000
MGSNSIVSNRWMSEKMNCLDLSPNSNSDNEEEEVHVLAVDDSFVDRKVIERLLKISSCKVTAVDSGWGALKLL
GLLDDEEEDKSSSSSSSSSSAGFEGLKVDLIITDYCMPGMTGYELLKKIKESSSFREIPVVIMSSENVMARIDRCLE
EGAEEFIAKPVKLSDVKRLRDYYMATREIRSVQSSSNVNKRKLQESFDVSTSSSPPSISPSPSPSSASPPSLFSSSAP
CSPSSLDSPTRRIKMTSFD
>Potri.002G082200
MEIMESGVVDTQHQEEEKHQQQKEEEGEEKHKQQRDQEGEEEEKHFHVLAVDDSFIDRKLLERLLKVSSYQVT
FVDSGDKALEYLGLLDSIDNVNATSSSSSSQSPQQEGMKVNLIMTDYCMPGMSGYDLLKRVKGSYWKDVPVV
VMSSENIPSRIRMCLEEGAEEFLLKPLQLSDVEKLQTHLLKSLDKYSSKRIDDNSFNTDSSNITVSNKSNSNNIVSK
RKALSPEIEDRRPKMKGLAVV
>Potri.003G197500

MIITDYCMPGMTGYDLLKKIKESKYFKDIPVVIMSSENVPSRINRCLEEGAEEFFLKPVQLSDVNKLRPHLMKGR
CKEEDQPNNKRKGMEEIVNSPGRTRSRYNEGLEVVLSQ
>Potri.001G027000
MAVEMALATTMATETQFHVLAVDDCLIDRKLIERLLKTSSYQVTAVDSGSKALEFLGLNGENELRDSKPASVSPD
PYHQHIEINMIITDYCMPGMTGYDLLKKIKESKYFKDIPVVIMSSENVPSRINRCLKEGAEEFFLKPVQLSDVNKL
RPHLMKGRCKEEEEEEEEDQPNNKRKGMEEIVNSPDRTRTRYNDGLEVV
>Potri.006G041100
MTITGDSHSQFHVLAVDDSMIDRKLIERLLKTSSYQVTAVDSGSKALKFLGLHEEDDHSNPDTVPSVSPNDHRE
VEVNLIITDYCMPGMTGYDLLKKVKESSSLRDIPVVIMSSENVPSRITRCLEEGAEEFFLKPVRLADLNRLKPHM
MKTKIKNEKQEDQEKLENSEIQSEQQPQPPSQPQPQPQPQLESQSQPQPSPLLQQPNNNKRKAMEEGLSPD
RTRPRYNGITTMV
>Potri.016G038000
MAITGDSLSQFHVLAVDDSLIDRKLIERLLKTSSYQVTTVDSGSKALKFLGLQEDEQSNPDTPYVSPNNHQEME
VNLIITDYCMPGMTGYDLLKKVKESSSLRNIPVVIMSSENVPSRITRCLEEGAEEFFLKPVRLSDLNRLKPHMMK
TKIKNQKQEEQEELEIPAIQSEEQKQPVQQPSSQPQPESQPQPSALLLQPNNNKRKAMEEGLSPDRTRPRYNGI
TTMV
>Potri.019G133600
MNGHVWIAYLKPYNFLSFFLKVSFSFCVLLRWLDIVVCSLKVDMASSSSSLPSMEFDFDEKPHVLAVDDSLIDRK
VIERLLINSTCRVTTAENGKRALEYLGLADGQHPSHSQDLKVNMIITDYSMPGMTGYELLKRIKESPTMKEIPVV
VVSSENIPTRINQCMEGGAQEFLLKPLQLSDATKLRCHIKKLNN
>Potri.015G070000
MATAGEILRRSLTEEVGFSKGSVSGSEELHVLAVDDSFVDRKVIERLLKISSCKVTVVESGSRALQYLGLDGEKSSV
GFNDLKINLIMTDYSMPGMTGYELLKKIKQESSAFREIPVVIMSSENILARIDRCLEEGAEEYILKPVKLSDVKRIKD
VIMGGDGEQKKRRSVRKRGREDCFYSLSQPQLVQSSSSPAFDFLPSELQSSSPFSTLSLSKRPKLQTRD
>Potri.008G213500
MMNLANCKGSMSTATSGGVWKASDGASDQFPAGLRVLVVDDDPTCLVILEKMLRTCRYEVTKCNRAEIALSLL
RENKNGYDIVISDVHMPDMDGFKLLEQIGLEMDLPVIMMSADDGKNVVMKGVTHGACDYLIKPIRIEALKNI
WQHVVRKRKNEWKDLEQSGSVEEGGDRQQKQPEDADYSSSANEGSWKNSKRRKDEEEEAEERDDTSALKK
PRVVWSVELHQQFVAAVNQLGIDKAVPKKILELMNVPGLTRENVASHLQKYRLYLRRLSGVSQHQNGMGSSFI
SPQEATYGPLSSLNGLDLQTLATAGQLPAQSLATLQAAGLGRSTAKPRMPMPIVDQRNLFSFENPKLRFGEGQQ
QHLNNGKQTNLLHGIPTTMEPKQLANLHHSAQSLGSMNMQFNAHAGQSSSLLMQMSQQQSRGQILNETT
HSHVPRLSSSIGQPIASNALASGVLTRNGLAENGRGIGFNPVSQSSTLLNFPLNTTAELTATSFPLGSAAEVPSLTS
KGTFQEEISSEIKGTGGFMPSYDIFSDLQQHRSHDWELQNVGMTFNGSQQSNSVQSNLDVAPSVLSHQGFSS
CQSNGQSRNNISVVGKPMFSAGDATEHVNAQSLEHPINTFFAENSMRVKTERVPDANPQTALFNGQFGQED
LMSALLKQQQQQQQGGIGLAENEFDFDGYSLDNIPV
>Potri.019G058900
MDGGGGSRENVMVGGAYTEEPHVLAVDDSLVDRKLVERLLKNSSCKVTTAENGLRALEYLGLGDEKRTSLEDN
VSKVNLIITDYCMPGMTGYELLKKIKESSMLKEIPVVIMSSENIPTRINKCLEEGAQMFMLKPLKQSDVVKLRCNL
MNCRS
>Potri.010G001000
MLNLGYCKGSMSTASSGGVSDQFPAGLRVLVVDDDPTCLVILEKMLRTCLYEVTKCNRAEIALSLLRENRNGYDI
VISDVHMPDMDGFKLLELIGLEMDLPVIMMSADDGKNVVMKGVTHGACDYLIKPIRIEALKNIWQHVVRKRK
NEWKDLEQSGSVEEGGDRQQKQSEDADYSSSANEGSWKNSKRRKDEEEEADERDDTSTLKKPRVVWSVELH
QQFVAAVHQLGIDKAVPKKILELMNVPGLTRENVASHLQKYRLYLRRLSGVSQHQSGMGNSFINPQEATYGPLS

SLNGLDLQTLAAAGQIPAQSLATLQAAGLGRSTAKPRMPMPIVDQRNLFSFENPKLRFGEGQQQHLNNGKQI
NLLHGIPTTMEPKQLADLHHSAQSLGSMNMQLNAHGDQSGQSGSLLMQMSQQQSRGQILNETTSSQVPRL
PSSIGQPIVTNAIASGVLARNGLAENGRGTGFNPVSQSSPTLNFPLNTTAELTATSFALGSAPGVPSLTSKGTFPE
DISSEMKGPGGFIMPSYDIFSELQHHRSHDWELQNVGMAFNGSQQSNSLQSNVDVASSVLAHQGFSSSQSN
GQGRNISAVSKPIFSAGDATSHVNAQSLGQPLNTFFAENLVRVKTERVPDANLQTTLFNEQFGQEDLMSALLKQ
QQQAGIGPAEIEFDFDGYSLDNIPV
>Potri.008G181000
MMENNGFSSPRNDSFPAGLRVLVVDDDPTWLKILEKMLKKCSYEVTTCGLARDALNLLRERKGGYDIVISDVY
MPDMDGFKLLEQVGLEMDLPVISRFFSLTLEFYFNANIFYLRIHIPMSCLHYIYIGILIQLLTSCACQLTVMSVDGE
TSRVMKGVQHGACDYLLKPIRMKELRNIWQHVFRKKIHEVRDIETLERVESFQMTRNGSDQYEDGHVLCGEDL
TSIKKRKHIESKHDEKDTGDSISTKKARVVWSVDLHQKFVKAVNQIGFDTTSLPSFDGAMTGSVCLAEVGPKKIL
DMMNVPWLTRENVASHLQKYRLYLSRLQKENDFKNPAGGIKQSDSPLRDSAGSFGSQNSINLQHNDVSNGSY
RFSGSSLVVHNGDPRSHDSDRKRLVSTPVEEPKRTLTVNVPNPCIPRSSQMEFGHPLAPPQSEVDFGALDSTFP
TKYPWCGIPEIQLKKEHNPLHLNDEFSHLPLPGQKQLTQADYPQPAPAISSAPSLTPSHNEYRSNVNHASSTAIAV
DSSPIQTKTNVANHQAIELISKSTPSLENQGFNMNSITEFESSRKNINLGMLPFTTLEEDLQVCWVPGDCYMNL
GLQNIEVLEYFDPGLITDVPVNLNDGLRFDYEFNDPTEYSLIDQSLFIA
>Potri.008G135500
MAALQRVAPSLGTSASTYGSCKGAGADVIVSDQFPAGLRVLVVDDDITCLRLLEKMLRRCLYHVTTCSQATAALK
LLRERKGCFDVVLSDVHMPDMDGFKLLELVGLEMDLPVIMMSADGRTSAVMRGISHGACDYLIKPIREEELKNI
WQHVIRKKWNENKEQEHSGSFEDNDRHKRGNDAENASSVSEGAEGVLKGQKKRSIAKDEDDAELENDDPSA
SKKPRVVWSVELHQQFVSAVNHLGIDKAVPKRILELMNVPGLTRENVASHLQKFRLYLKRLSGVAQQGGISNPF
CGLLDSNVKLNPLGRFDIQVLAASGQIPPQTLAALHAELFGHPAGSMVTTVDQPALLQASVQGPKCIPAEHGM
AFGQPLVKCQTNMSKHFPQNVVSAEEVATGFVAWPSNSVGTVGPISNLGGMSSKNSNMLMDVVQQQKQQ
QQSPQLPSLPEPSRIINVQPSCLVVPSQSSATFQAGNSPASVNQNCSYSRSPMIDYSLLSPQSNNSSLNIGQISNG
DLKTTGVSGYSGSGSISPLSSCSVNPDNTSQQGQSSTMTIRAARQLPGLGPNISNFQGSYGAKSSEVLDQGPLR
NLGFVGKCTSIPSRLAVDEFESTMSNLEHEKLYMETNGNKVKQEPNSNAVKQEPNVEFMDNAKVLIPILPQLPP
NTSDLMSVFKE
>Potri.010G105600
MAALQRVASSLSTSASSYGSCKGAGADLMFSDQFPAGLRVLVVDDDITCLRLLEKMLCRCLYNVTTCSQATAALK
LLRERKGCFDVVLSDVHMPDMDGFKLLELVGLEMDLPVIMMSADGRTSAVMRGIRHGACDYLIKPIREEELKN
IWQHVIRKKWNENKEQEHSGSFEDNDWYKRGNDDTENASSVNEGAEGVLKGQKKRNIAKEEDDNELEIDDP
SASKKPRVVWSVELHQQFVSAVNQLGIDKAVPKRILELMNVPGLTRENVASHLQKFRLYLKRLSGVAQQGGISN
TFCGPLDSNVKVNPLGRFDIQALAASSQIPPQTLAALHAELFGRPTGSLVTTVDQPALLQASMQGPKCIPVEHG
VAFGQPLVKCQTNISKHFPRNVISVEDVASGFGAWPSNSVGTVGSSSNLGGMNSQNSNMLMDILQQQKQQ
QQPLQQSSLSEPSRSINVQPSCLVVPSQSPATFQAGNSPASVDQNCSYNRSTMIDYSLLSAQSNNSSLNIGQILN
GDLKTTGVSGYSGSVSISPLSPCSVDPDNTSQQGRSSTMAIRAVRQLPGLVPNISNLQGSYSARSSEVLDQRPLR
NLGFVAKCTSIPSRLAVDEFESTMSSLDHEKSYMEISGNKVKQEPNIEYMDNARVGIPILQQLPPAPSDLMSVFT
E
>Potri.012G133800
MASTQSSNALSSSLPKIHILIVDDDSTSLSVVSATLKTVSYKDNRNLELIVVTVKKPFDALSILRLKKGLFDLVVSDL
HMPEMNGMELQKQVEEEFKLPVIIMSSDESKNVISRSLEGGAAFYIVKPANKVDLKNVWHYAVAIKTGNSLSIK
EIEGSREPSYSSTLVERLSLEDVNSATSINDEKRYRKNGRKEERKSTKDDQEVDSQPASKKPKVVWTNSLHNRFLL
ALNHIGLDKAVPKRILECMSVRGLSRENIASHLQKYRIFLKKVAERGGCSSKNLSGRDDLRTNFAPSQPFMMFK
NVQQEYSQRVTRASIQPGLGGNTIPALGGSSFRNLHSPNQQAPSNNSILQSPYGQSRLFGNANSANQANQIRS

GLESNYSIGENMPSYGGVIPNGLTHGTRPMQMYHPQNQARSYLQNFGSPPLINRFGAVGIQATNYGSGVGDI
GIINNSLNTNNNYVGIRVTTDGRLIGSGQMQLNTNELSNGFSDGGHPCLMNWARNGNMNVASIGFNIASEN
YVARRGSFSAGFEGANQFFPPTPFTSVYHGNSTPMLLPPPPPQHQLGLGNGGQNDFVFGLMNNSPPILGGNS
NQQPQIGEGEPNVSDMLLEPTNNPPAYQLPQGVAQMSTPLHELLTPDFLNSLHIDDNTPRNEQPSSQA
>Potri.006G262100
MTVEQGIGDSNIDQFPIGMRVLAVDDDPTCLLLLETLLRRCQYTVTTTSQAITALSMLRENKNKFDLVISDVHM
PDMDGFKLLELVGLEMDLPVIMLSANGDPKLVMKGITHGACDYLLKPVRIEELKNIWQHVIRRRKYDNKDRNS
SDNRDKPNQGSSEAIPDQKLNKKRKDQNEDEDEDRDENEHENEDPATQKKPRVVWSVELHRKFVAAVNQLGI
DKAVPKKILDLMNVEKLTRENVASHLQKYRLYLKRISTVANQQANMVAALGSSDASYLQMNSMNGMGLHNL
AGSGQFHNTPFRSLPSSGMLSRLNSPAVLGIRGLPSPGVIRLGHVQSAPHSANGLSHFQPVGHPGNNGNILQG
MPMSLELDQIQSNKGVNYVRELPTHLDDTASFPVSSGSTDMKIIAGSSNSPFVGVSGKPLMLEGHGQGLQDG
QKFGNQSSLAAGSLDPGYSSHFPDHGRCNDNWSNAVQSNGVQSSSFTLNDCFKQSTLHPSSIRDSMSTMAL
QSRNNHCDVSSISTLPIHLQDSKADLQCQVATMSSDAGQIINNGQLGWDDHRQDDPYHANGISNSINSAIPIN
GNGSLVGQSLDPNNMIFQRTRSFSSAGQSNFVDTSLMKHNEVESSAMETLVRSKDGYLLGQQKQQDSYVSN
NFGSLEDLVSVMVKQEQDKVKLPEGDFGCGGYSLRTCI
>Potri.018G111300
MTVEDQRGCNSVNEKMFPVGMRVLAVDDDPICLKVLENLLRKCQYEVTTTNQAVTALEMLRENRNKYDLVIS
DVNMPDMDGFKLLELVGLEMDLPVIMLSSHGDKEFVFKGITHGAVDYLLKPVRLEELKNIWQHVIRRKKWYP
QDQNGSPDQDKGGDGAGEGEQVTSTGSADQNGKVNRKRKDQDEEEGEGEDGNDNEESGNQKKPRVVW
SVELHQKFVSAVNQLGLDKAVPKKILDLMNVDGLTRENVASHLQKFRLYLKRLSCGANQQPNMVAAFGAKDSS
YLRMGSLDGFGDFRSVHGPGQLSTTSLSSYPPGSLLGRLNSPGGLTLQGIASPGLLQPGHSRSLNNPLNTLGKLQ
PGLLQTNRGSTNLFQGISSALDPKQFQLKSMNHTGDFNHKGDATSFTLAGCFPDVKVTIGSLGNTISSAANNPQ
MLHVNPQQNQTRRSLATQSSLSMPSLNQESFDVGVRGSSNFLDHSRCDDNWQGVVQMSTFPSNSLPLGEPF
RHDPLPTSTRRDNISSTTSHIVNNPLDFSSSGSLTAPLEDSRLDMQGQADLVGNIFHNTNYTSKNRWGENSQNF
NPCLNGSFGAMNSLVSGNGSMNPLSQSMDQRKRFDASVLGQSNSGTLSMFQHLEAGNSALDPTGMRIPKM
RSNEDFLLEQTKSPNGFVQNNYDSLDDIVNAMIKRDQNDGVLMDGEFGFESYSPGSCI
>Potri.015G136000
MASSQSSNIIQNSSLLKIKILVVDDDSTSLSIVSAMLKTCSYKENRKLELFVVTVKNPFDALSTLRLKKGLFDLVVTD
LHMPEMNGMELQQQVDEEFKLPVIIMSSDDSEKVILRTLEGGAAFYIVKPINKDDLKNVWQYAVATKTGKYSLS
IKEIGGSQESSSSTVFDQKISVDEVSSAKSTNIKICNEKDRINKNRKRTKEDQEVDSQLAPKKPKVVWTNSLHSRF
LQAINHIGLDKAVPKRILEFMSVPGLSRENVASHLQKYRIFLKKVAERGTSSSKNLSGRALKSNFASSQPSLMLKN
FQQEYSQFSRQQQVLRASVQAGFGGNTIPTPDGGSNFGSLHSPNQQAPNNNSALQSPYKQSRSFGNPTGFQ
QLSFGNANQANKKRSGLESNNNTGTNPPASRESVPNGLTHGKSPMQIYQPQNQGRSNLQSFDSSEHNKFGSI
GVQATNYRSGVGNIGNNNNSLNTNNNYAGIRVNTDGQLIGAGQLQVSKNKLSNGFGNGGNCGLMNWTHN
GNMNVASSSYLAQRGSFPASIESANQFPPTLFTSTVYQGNTPMLLPPPPPPPQQQQQLGLGNGGQHDFAFGL
VNNSPPIFGGNYNQQQQFGQGELNFSDMLLEPTNNLTAHQPQGVDQVSTPLHDLLHPDFLNSLHIDDNTPW
NEQPSSQVQSGEEAMNPFFGGNASSMDGYFPNFNQASVRHTNIAALILSIVFTISNN
>Potri.010G053100
MMENGFSSPRNDSFPAGLRVLVVDDDPTWLKILEKMLKRCSYEVTTCGLARDALNLLRERKGGYDIVISDVYM
PDMDGFKLLEHVGLEMDLPVIMMSVDGETSRVMKGVQHGACDYLLKPIRMKELRNIWQHVFRKKIHEVRDI
EILEGIESFQMTRNGSDLYEDGHFLCGEDLTLIKKRKDIESKHDEKDIGDNTSAKKARVVWSVELHQKFVKAVNQ
IGFDKVGPKKILDLMNVPRLTRENVASHLQKYRLYLSRLQKGNDFNNPVGMIKQSDSPLSDSAGSFGSQNSINL
QPTDVSNGCYGFSVSSLVLHNVDPRSHNGDRKILVTTPVAEPKRGVTVDAPNPRKPRISQMEFGHPLAPHFND
GFSHLPMPGQKQLTRADYPQPARAISSAHSLTEREIGCPVKIKPSRNDYGSNASHVSSTANAVDSIPLQTKTNST

NHQVSSTSSMENQGLNMNCLTDVESSRKNINLGMPPFAPLDEDLQVRFVPGDYYMNLGLQNIEVPEYFDPSLL
TDVPIHLNDGLRFDYEFYDPTEYSLIDQSLFI
>Potri.018G021300
MTVEQGIGDSNIDQFPIGMRVLAVDDDPTCLLLLETLLRRCQYNVTTTSQAITALRMLRENKNKFDLVISDVHM
PDMDGFKLLELVGLEMDLPVIMLSANGDPKLVMKGITHGACYYLLKPVRIEELKTIWQHVIRRKKSDNKDRNSS
DNRDKPNQGSSEAVPDQKLNKKRKDQNGDEDEDHDEDEDEHENEDPTTQKKPRVVWSVELHRKFVAAVNQ
LGVDKAVPKKILDLMNVEKLTRENVASHLQKYRHYLKRISTVANQQANMVAALGSSDASYLQMNSMSVLGLH
SLAGSVQFHSTPFRSLQSSGMLDRLNSPAVLGIHGLPSPGVIQLGHVQTAPHTANGLSHFQPVGHRGNNGNIL
QGMPMPLELDQIQSNKGVNYIPELPTHLDDTASFPVSSGSTDMKIIAGSSNSPFVGVSNKHLMLEGHGQGLQ
DGQKSGKQSSLSAGSLNPGYSSHFPDHGRCNDNWSNAVPSNGAQSDSFTLNDYFKQSTLHPSAIRDRMSTM
ALQSRNNPCDVSSVSTLPMHLQDSKADLPCRVGVATVSSNAGQLINNGSLGWDDRRQDDPYHSNGLSNSINS
AFPINGNGSPNGFNLDPNNLFFQRTTSFISTGPSNFVDTSLMKHNEVECSAMETLVRSKDGYLLGQEKPQDSSV
SNNFGSLEDLVSVMINQPTILG
>Potri.006G188000
MMAVDDQRGGNSVNEKKFPVGMRILAVDDDPICLKVLENLLRKCLYEVTTTNQAVTALEMLRENRNKYDLVIS
DVNMPDMDGFKLLELVGLEMDLPVIMLSSHGDKEFVYKGVTHGAVDYLLKPVRMEELKNIWQHVIRRKKWY
PEDQNRSPNQDKGDDGAGEGEQGVTSTGSADQNGKLNRKRKDQDDEDEEEGEDGDDNEVSGNQKKPRVV
WSVDLHQKFVAAVNQMGLDKAVPKKILDLMNVDGLTRENVASHLQKFRLYLKRLSSGGNQQGNMVSAFGAK
DSSYLQMSSVDGFGDFRSMHGPGRLSTTSLSSYPSGTFLGRLNSPGGLTLQGIASSGLHQPGLLPTNQGTNLFQ
GISSALEPKQLQMKSINHIGEFNQKGDTAGFTLASCFPDVKVTIGSLSNTISGATSSPPILQANPQQNHNRGSLVT
QSSLSMPSLNQESFDVGVQGSSNFLNHSRCDDNWQGAVQLSTFPSLPIGEPFRHDQLPSSTLRGNISSTTCHIV
NSPIDFSSTGSLTAPLEDSRVDMQGQTDFIGNIFQNTTNYISKNRWEENSQNYNPGLNGSFGAMNSLVSGNGS
SGALNQSMDQRKRFGASMIGQSNSGTLSMFQHPDVENFALDPKMRSNEDVILAQTKSQNGFVQNNYESLD
DIMNEMIKQDQNDGSLMDGEFRFGSFSPGSCM
>Potri.018G094700
MAKNNDEVSEISMHDEGRGDMFNPADLRVLAIDANVVCLKYLVAILQKCQYRVTSTTLAAEALKMLRENKNDY
HVVITDVKRLDMDGFKLLEIIGLEMDLPVILVSAEDSQSSIMKGIRHGARDYLLKPVRIQEMQNIWQHVVRKRL
SDSSDKSSSFTKEIIEQESVPSTEPMAEAEVTGVKEKEIIGNPNGDACSGKKPRVTWSSELHVKFVDCVEKLEAR
GERVQPKRIREMMNVEGLSRENIASHLQKYRNLLKKHKDKMNQQDNAGTDLNRYGRISATKNGLWNTYGDS
TPKPQSLASSCTDSKRMFFQPSKQNVFLENSLSSNITMRKSVPVPEFKGLNFSASSSTWELPTEYTGSQSFGQN
PGLCNLSPLLCSPAASFKPSFPMLGQTLIDRSPASVLSNPSYFPGQYGEGISMNLCGSGTSNFREHDSHCAYRDQ
KSLLEDFMNSEEAKLFMDEEISDMVNQAIVFFRLTKWQFDISPLIMVQTCSTVRQNHNTSFSSPLQFNSDGTPR
L
