# Supplementary material for: Genome-Wide Two-Component System Genes of Cytokinin Analysis and Their Functions of Drought Stress Response in Pinus tabuliformis
Source: Metabolites. 2026 Oct 1;16(10):741. doi: 10.3390/metabo16100741 (PMC13707277; doi:10.3390/metabo16100741)
Supplement: Supplementary file 1 [file metabolites-16-00741-s001.zip › Supplementary Table S4.pdf]

Supplemental Table S4. Prediction of subcellular localization of PtTCSs protein.

Gene name	subcellular localization	Gene name	subcellular localization
PtCHK1	plasma membrane	*PtRR5*	nuleus
PtCHK2	plasma membrane	*PtRR6*	nuleus
PtCHK3	plasma membrane	*PtRR7*	nuleus
PtHP1	cytoplasm	*PtRR8*	cytoplasm
PtHP2	cytoplasm	*PtRR9*	nuleus
PtHP3	mitochondrion	*PtRR10*	nuleus
PtHP4	chloroplast	*PtRR11*	nuleus
PtRR1	nuleus	*PtRR12*	cytoplasm
PtRR2	nuleus	*PtRR13*	cytoplasm
PtRR3	nuleus	*PtRR14*	cytoplasm
PtRR4	nuleus	*PtRR15*	nuleus
